# Associations of per- and polyfluoroalkyl substances (PFAS) and their mixture with risk of rheumatoid arthritis in the U.S. adult population

**DOI:** 10.1186/s12940-024-01073-3

**Published:** 2024-04-13

**Authors:** Jian-Chao Qiao, Zhen-Hua Li, Yu-Bo Ma, Hui-Ya Ma, Meng-Yue Zhang, Xiu-Jun Zhang, Cheng-Yang Hu

**Affiliations:** 1 Department of Clinical Medicine, The Second School of Clinical Medicine, Anhui Medical University, 81 Meishan Road, Hefei, 230032 China; 2 Department of Epidemiology and Biostatistics, School of Public Health, Anhui Medical University, 81 Meishan Road, Hefei, 230032 China; 3 Management & Checkup Center, the First Affiliated Hospital of Anhui Medical University, 218 Jixi Road, Hefei, Anhui 230022 China; 4 Key Laboratory of Population Health Across Life Cycle (Anhui Medical University), Ministry of Education of the People’s Republic of China, 81 Meishan Road, Hefei, 230032 China; 5 Department of Environmental Medicine and Public Health, Icahn School of Medicine at Mount Sinai, One Gustave L. Levy Place, Box 1057, New York, NY 10029 USA; 6 Department of Humanistic Medicine, School of Public Health, Anhui Medical University, 81 Meishan Road, Hefei, 230032 China

**Keywords:** Rheumatoid arthritis, Per- and polyfluoroalkyl substances, PFAS, Mixed effect

## Abstract

**Background:**

Per- and polyfluoroalkyl substances (PFAS) are known environmental contaminants with immunosuppressive properties. Their connection to rheumatoid arthritis (RA), a condition influenced by the immune system, is not well studied. This research explores the association between PFAS exposure and RA prevalence.

**Methods:**

This research utilized data from the NHANES, encompassing a sample of 10,496 adults from the 2003–2018 cycles, focusing on serum levels of several PFAS. The presence of RA was determined based on self-reports. This study used multivariable logistic regression to assess the relationship between individual PFAS and RA risk, adjusting for covariates to calculate odds ratios (ORs). The combined effects of PFAS mixtures were evaluated using BKMR, WQS regression, and quantile g-computation. Additionally, sex-specific associations were explored through stratified analysis.

**Results:**

Higher serum PFOA (OR = 0.88, 95% CI: 0.79, 0.98), PFHxS (OR = 0.91, 95% CI: 0.83, 1.00), PFNA (OR = 0.87, 95% CI: 0.77, 0.98), and PFDA (OR = 0.89, 95% CI: 0.81, 0.99) concentration was related to lower odds of RA. Sex-specific analysis in single chemical models indicated the significant inverse associations were only evident in females. BKMR did not show an obvious pattern of RA estimates across PFAS mixture. The outcomes of sex-stratified quantile g-computation demonstrated that an increase in PFAS mixture was associated with a decreased odds of RA in females (OR: 0.76, 95% CI: 0.62, 0.92). We identified a significant interaction term of the WQS*sex in the 100 repeated hold out WQS analysis. Notably, a higher concentration of the PFAS mixture was significantly associated with reduced odds of RA in females (mean OR = 0.93, 95% CI: 0.88, 0.98).

**Conclusions:**

This study indicates potential sex-specific associations of exposure to various individual PFAS and their mixtures with RA. Notably, the observed inverse relationships were statistically significant in females but not in males. These findings contribute to the growing body of evidence indicating that PFAS may have immunosuppressive effects.

**Supplementary Information:**

The online version contains supplementary material available at 10.1186/s12940-024-01073-3.

## Introduction

Rheumatoid arthritis (RA) is a chronic autoimmune disorder characterized by inflammatory polyarthritis, predominantly affecting the smaller joints. It is estimated to affect between 0.24 and 1% of the global population, exhibiting a prevalence approximately twice as high in women compared to men [1]. The articular and systemic manifestations of RA have the potential to culminate in severe long-term consequences, including disability and mortality [2]. The etiology of RA is multifactorial, likely stemming from an intricate interplay between genetic susceptibilities and diverse lifestyle determinants [3–5]. Previous scientific investigations suggest a potential link between RA and exposure to environmental contaminants [6–9]. Notably, the influence of per- and polyfluoroalkyl substances (PFAS) in this context appears to be an underexplored avenue.

PFAS are a group of synthetic fluorinated organic compounds widely used in various consumer and commercial products, encompassing items such as upholstery, carpeting, clothing, nonstick kitchenware, firefighting foams, and specialized food packaging [10]. The recent meta-analysis that estimated the half-lives of PFAS in human studies found that the mean half-life ranged from 3.4 to 5.7 years for total perfluorooctanesulfonic acid (PFOS), from 1.48 to 5.1 years for perfluorooctanoic acid (PFOA), and from 2.84 to 8.5 years for perfluorohexanesulfonic acid (PFHxS) [11]. Due to their pervasive application and remarkable environmental persistence, human exposure to PFAS is virtually inescapable, predominantly stemming from dietary sources, potable water, and a mix of indoor and outdoor environments [10, 12, 13].

Previous research has consistently demonstrated an association between PFAS exposure and a wide array of adverse health outcomes. These include, but are not limited to, dyslipidemia, hypertension, diabetes, metabolic syndrome, various cancers, and negative pregnancy outcomes [14–19]. The existing evidence from animal studies indicates potential disruptions in immune functions as a consequence of exposure to PFAS [20–22]. In addition, human epidemiological studies have further explored the potential associations of PFAS exposure with various immunological outcomes [23–28], encompassing asthma, allergic manifestations, susceptibility to infectious agents, and serological responses after vaccination. Cumulatively, the existing body of evidence suggests that PFAS exposure might exert effects similar to the onset and persistence of RA, which is an immune-related disease.

In the existing literature, investigations into the relationships between PFAS exposure and RA are notably limited. Two previous studies [29, 30] have assessed the association of RA with PFAS exposure among population exposed to extremely high environmental concentrations, and one [29] of them reported that RA was positively linked to PFOA exposure among workers. One recent study from China among the general population suggested that specific PFAS exposure is associated with alterations in defined immune markers of RA [31]. In addition, results from two other studies conducted in China indicated that certain types of PFAS exposure may either be associated with an increased risk of RA or enhance the disease activity of RA [32, 33]. While comprehensive data clarifying the association between PFAS exposure and RA risk remains scarce, various studies have underscored a potential association between RA risk and exposure to environmental contaminants, including but not limited to heavy metals [9], phthalates [7], and airborne pollutants [34]. Given the hypothesized shared pathogenic mechanisms, it becomes imperative to systematically assess the association of PFAS exposure with the risk of RA. Based on a comprehensive consideration of multiple factors, an initial hypothesis was formulated that elevated PFAS exposure correlates with an increasing risk of RA. In light of this, our study aims to clarify the association of serum PFAS concentrations, both as individual and mixtures, with RA risk, leveraging a robust cross-sectional study design and, explore if these associations differed by sex.

## Methods

### Study population and design

The National Health and Nutrition Examination Survey (NHANES) is an ongoing, nationally representative survey that utilizes a complex multi-stage sampling methodology. This open-access initiative, carried out across the United States, aims to capture the nutritional and health profiles of the non-institutionalized U.S. population (National Center for Health Statistics). The survey is conducted biennially.

**Fig. 1:**
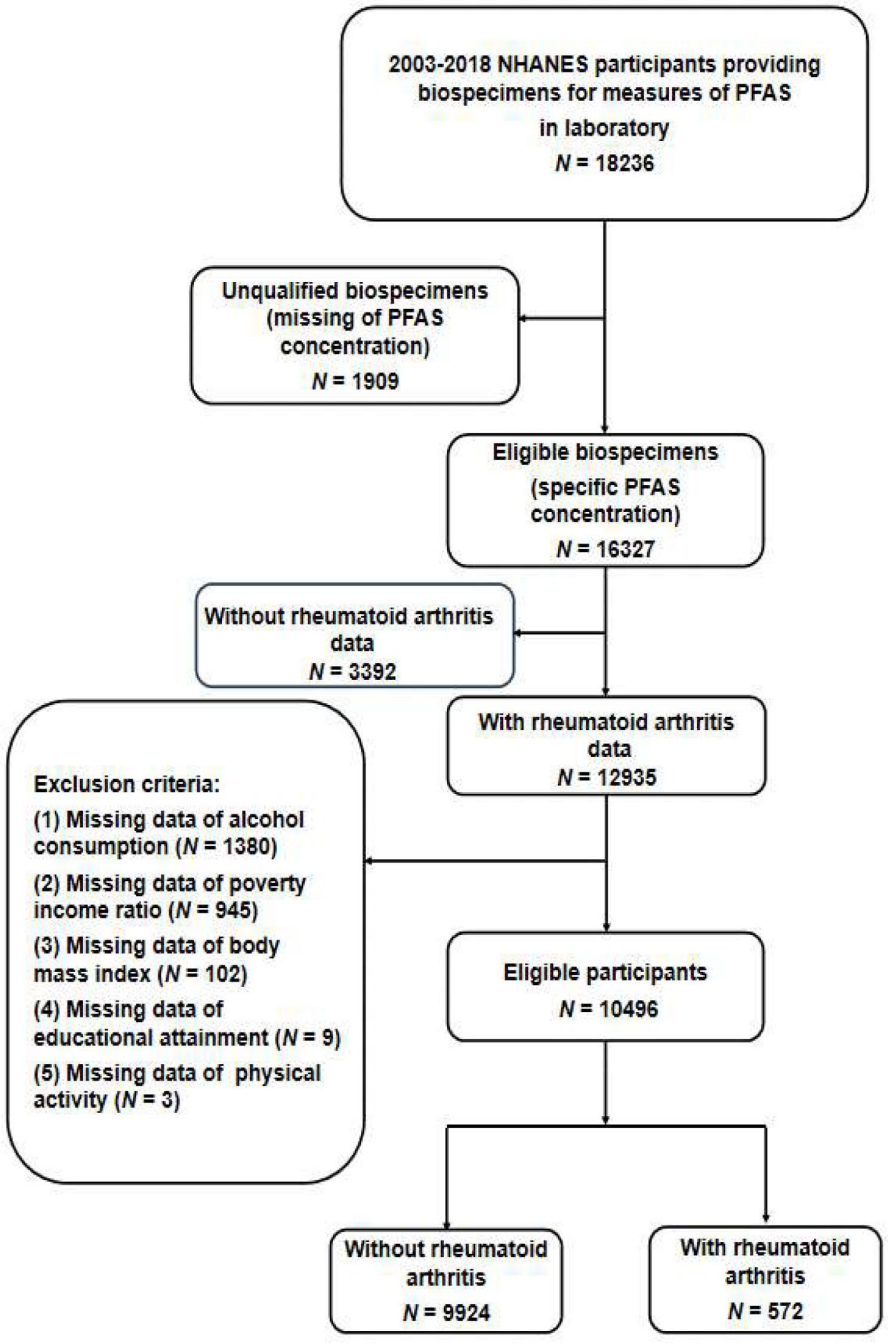
Flowchart of participants selection from the NHANES 2003–2018

Between 2003 and 2018, NHANES registered a total of 76,848 participants. However, 58,612 of these participants were excluded as they did not provide biospecimens for PFAS measurements. Furthermore, 1909 participants were excluded due to unqualified biospecimens, which led to missing PFAS concentration data, often resulting from the provision of insufficient or substandard plasma samples. Another 3392 participants without RA data were also excluded. Figure 1 shows the detailed participant selection process. Every participant submitted written consent, and the data gathering approach, along with the research protocol, received approval from the National Center for Health Statistics (NCHS) Research Ethics Review Board (protocol number: #98-12, #2005-06, #2011-17, #2018-01) [35]. To maintain analytical consistency, we excluded participants lacking available covariate data (*N* = 2439). Finally, this study comprised 10,496 individuals, consisting of 9924 without RA and 572 with RA.

### Exposure assessment: serum PFAS concentrations

Serum samples collected from participants were processed and stored at − 80 °C in specialized containers and subsequently transported to a CDC (Centers for Disease Control and Prevention) designated laboratory for analysis. Detailed procedures for specimen collection and processing are outlined in the NHANES laboratory/medical technician procedure manual [36, 37]. For the quantitative detection of PFOA, PFOS, pefluorodecanoic acid (PFDA), PFHxS, perfluorononanoic acid (PFNA), perfluoroundecanoic acid (PFUnDA), 2-N-methyl-perfluorooctane sulfonamido acetic acid (Me-PFOSA-AcOH), online solid phase extraction coupled with High Performance Liquid Chromatography-Turbo Ion Spray-Tandem Mass Spectrometry (online SPE-HPLC-TIS-MS/MS) was employed. The limit of detection (LOD) for each PFAS was set at three times the standard deviation (SD) of the blank concentration [38]. In instances where the concentration was below the LOD, the machine-read value obtained through machine reading from the instrumental analysis was utilized if detectable. In cases where no machine-read value was available, the values were imputed using LOD/$\sqrt{2}$ [39].

### Outcome assessment: rheumatoid arthritis

The determination of participants’ RA status or non-RA status was achieved through a structured questionnaire. Initially, participants were posed the question, “Has a doctor or another medical professional ever diagnosed you with arthritis?” In cases where the response was affirmative, a subsequent question was raised: “Which specific type of arthritis were you diagnosed with?” Based on the responses to these two queries, participants were classified into either the RA or non-RA categories. Those who responded negatively to the primary question were allocated to the non-arthritis group, whereas individuals who answered positively to the first and identified “RA” for the second were categorized under the RA group.

### Covariate assessment

In our analysis, the selection of covariates was informed according to previous studies on the association of PFAS with RA [31, 32] and the using of a directed acyclic graph (DAG) (Supplemental Fig. S1). The categorical covariates encompassed sex (male, female), ethnicity (Mexican-American, non-Hispanic white, non-Hispanic black, other Hispanic, or other/multiracial), educational attainment (lower than high school, greater than or equal to high school), family poverty-income ratio (PIR) (≤ 1.30, 1.31–3.50, > 3.50), alcohol consumption (never, former, mild, moderate, heavy), and physical activity (yes, no). Age and body mass index (BMI) were used as continuous covariates for the comparation between the RA and no RA groups. In addition, age (young: 20–39, middle-aged: 40–59, old: ≥ 60 years) [7, 33] and BMI (< 25, 25–29.90, ≥ 30 kg/m^2^) [40, 41] were categorized for detailed characterization of the study population and to support subsequent subgroup analyses.

### Statistical methods

#### Individual pollutant analysis

We employed descriptive statistics to analyze the distribution of demographic characteristics, presenting the results as mean (± SD) or frequency (%) for the two groups (RA and non-RA). For variables that followed a normal distribution, we used the Student’s t test to compare between groups. In contrast, for variables with skewed distributions, the Wilcoxon rank-sum test was employed. Additionally, the chi-square test was used to assess differences in categorical variables across groups. To account for the skewed distributions of PFAS, we log_2_-transformed PFAS concentrations in subsequent analyses [42]. Pearson correlation coefficients among the PFAS biomarkers were calculated using log_2_-transformed concentrations. Our primary analysis involved multivariable logistic regression models to clarify the associations between PFAS concentrations and the odds of RA. These models were adjusted for a range of covariates: age, sex, ethnicity, educational attainment, BMI, PIR, alcohol consumption, and physical activity. Depending on the number of covariates, we employed three different models: unadjusted model was not adjusted for covariates; model 1 was adjusted for age and sex; model 2 was adjusted for all the covariates mentioned above. To address potential non-linear relationships, PFAS concentrations were modeled both continuously (as log_2_-transformed values) and categorically (in quartiles). In models where PFAS levels were treated continuously, the estimates reflected the change in odds of RA per doubling of PFAS concentrations. False Discovery Rate (FDR) correction using the Benjamini-Hochberg (BH) procedure was used to address the issue of multiple comparisons [43]. Statistical significance was determined with an FDR adjusted *p*-value (also called *q*-value) < 0.05, while results displaying an unadjusted *p*-value < 0.05 but an adjusted *p*-value > 0.05 were also discussed as marginal. All analyses were conducted using NHANES recommended sampling weights, which compensate for the survey’s stratified sampling design and non-response factors [44]. Subgroup analysis was also performed to evaluate the relationship between RA and PFAS in diverse populations by stratifying age, sex, ethnicity, educational attainment, BMI, PIR, alcohol consumption, and physical activity.

#### Mixture analysis

We examined the joint effects of seven PFAS on rheumatoid arthritis (RA) using three distinct approaches to model and parameterize exposure mixtures: quantile g-computation, Bayesian kernel machine regression (BKMR), and repeated hold out weighted quantile sum (WQS) regression. These models provide insights into both partial and cumulative dose-response relationships between environmental chemical mixtures and health outcomes, often obscured in single pollutant models. They also account for co-exposures that are highly correlated. All models were adjusted for age, sex, ethnicity, educational attainment, BMI, PIR, alcohol consumption, and physical activity. We also performed sex-stratified analyses for the mixture effect.

Our first approach, quantile g-computation, is a parametric, generalized linear model-based implementation of g-computation [45]. Quantile g-computation yields an estimate of the cumulative impact of the exposure mixture on the specified outcome, along with weights for each component within the mixture. These weights signify the proportional contribution of each element to the collective effect of the mixture. In this study, we specified quartiles as the quantile unit for PFAS concentrations. Thus, the mixture effect estimate denotes the OR of RA associated with a concurrent increase across quartiles in all six PFAS components. This method allows for both positive and negative effects of each exposure in the mixture, represented by relative weights summing to 1.0 [45].

The second approach, BKMR, uses a kernel function to flexibly model both the overall joint effect of an exposure mixture and to estimate individual exposure-outcome associations [46]. We conducted BKMR with 10,000 iterations, assessing convergence using the Markov chain Monte Carlo procedure. Univariate exposure-response functions evaluated the relationship between single PFAS levels and RA odds, while bivariate functions assessed interactions. We used posterior inclusion probabilities (PIPs) to determine the importance of each PFAS exposure [47]. The overall effect of the PFAS mixture was estimated by comparing the odds of RA when all PFAS exposures were set at the first and third quartiles versus the median value. We standardized all log_2_-transformed PFAS concentrations and excluded outliers exceeding 5 SDs from the mean (*N* = 8) [48].

The third approach, WQS regression, constructs a unidirectional weighted index from quantiled chemical exposures, addressing dimensionality and multi-collinearity issues in co-exposures [49], has been previously applied in several environmental epidemiologic studies [50–52]. We binned the chemicals into deciles, using negative weights from 100 bootstrap samples based on preliminary analysis suggesting PFAS were associated with lower RA odds. Significant chemicals of concern were determined as PFAS biomarkers with weights greater than a concern threshold of 1/c (14.3%), where c is the number of chemicals in the mixture as recommended by previous studies [49, 53]. We applied 100 repeated holdout validation to assess stability and potential generalizability [54]. WQS regression models with a WQS*sex interaction term and stratified sex-specific weights were also performed, allowing the WQS index effect to differ by sex [55]. Relative weights were calculated within each stratum, with chemicals exceeding 14.3% in at least 50% of the holdouts identified as chemicals of concern [56].

Sensitivity analyses in the individual pollutant model included adjusting for the NHANES calendar cycle. Multi-cycle analyses were also conducted to evaluate result stability. All statistical analyses were performed using R version 4.1.0 (R Core Team), with mixture analyses using the “bkmr”, “qgcomp”, and “gWQS” packages.

## Results

A total of 10,496 adults were included in the study, consisting of 5138 males and 5358 females. Among them, 572 participants had self-reported diagnoses of RA, resulting in a prevalence of 5.4%. The characteristics of the population, both with and without RA, are presented in Table 1. Noteworthy variations were observed in age, BMI, sex, ethnicity, educational attainment, PIR, alcohol consumption and physical activity.

**Table 1:** Baseline characteristics of participants by rheumatoid arthritis (RA) status, NHANES 2003–2018 (*N* = 10,496)

Characteristic	Overall	Without RA	RA	*p*-value
Age, mean (SE)	47.03 (0.29)	46.59 (0.30)	57.52 (0.83)	< 0.0001
**Age**, n (%)				< 0.0001
20–39	3578 (36.76)	3534 (37.83)	44 (11.33)	
40–59	3393 (38.23)	3194 (38.02)	199 (43.25)	
≥ 60	3525 (25.01)	3196 (24.15)	329 (45.42)	
BMI, mean (SE)	28.98 (0.11)	28.91 (0.11)	30.47 (0.40)	< 0.001
**BMI, n (%)**				< 0.001
25–30.0	3509 (33.49)	3350 (33.68)	159 (28.90)	
< 25	3020 (30.16)	2892 (30.40)	128 (24.51)	
≥ 30.0	3967 (36.35)	3682 (35.92)	285 (46.59)	
**Sex, n (%)**				< 0.001
Male	5138 (48.75)	4901 (49.20)	237 (38.26)	
Female	5358 (51.25)	5023 (50.80)	335 (61.74)	
**Ethnicity, n (%)**				< 0.0001
Non-Hispanic White	4860 (70.79)	4610 (70.91)	250 (67.93)	
Non-Hispanic Black	2147 (10.35)	1977 (10.05)	170 (17.50)	
Mexican American	1646 (7.62)	1563 (7.67)	83 (6.34)	
Other	1843 (11.24)	1774 (11.37)	69 (8.23)	
**Educational attainment, n (%)**				< 0.0001
< High school	2541 (15.13)	2357 (14.84)	184 (21.93)	
≥ High school	7955 (84.87)	7567 (85.16)	388 (78.07)	
**PIR, n (%)**				< 0.0001
≤ 1.30	3196 (20.26)	2960 (19.88)	236 (29.29)	
1.31–3.50	3968 (35.76)	3762 (35.62)	206 (39.13)	
> 3.5	3332 (43.97)	3202 (44.50)	130 (31.58)	
**Alcohol consumption, n (%)**				< 0.0001
Never	1488 (10.63)	1393 (10.51)	95 (13.50)	
Former	1866 (14.43)	1721 (13.99)	145 (24.93)	
Mild	3513 (36.60)	3322 (36.63)	191 (35.96)	
Moderate	1587 (17.38)	1524 (17.65)	63 (11.01)	
Heavy	2042 (20.96)	1964 (21.23)	78 (14.59)	
**Physical activity, n (%)**				< 0.0001
No	5077 (41.60)	4712 (40.80)	365 (60.62)	
Yes	5419 (58.40)	5212 (59.20)	207 (39.38)	

PIR: calculated by dividing family income by the poverty threshold

Alcohol consumption: “Never” < 12 drinks in lifetime, “Former” ≥ 12 drinks in 1 year and no drink last year, or no drink last year but ≥ 12 drinks in lifetime, “Mild” < 1 drinks/d for female and < 2 drinks/d for male, “Moderate”, 1 to 2 drinks/d is for female and 2 to 3 drinks/d is for male, “Heavy”, ≥ 3 drinks/d is for female and ≥ 4 drinks/d is for male

Physical activity: “Yes”, if engaging in moderate-intensity or vigorous-intensity sports, fitness, or recreational activities for > 10 min on a typical day; otherwise, “No”

*Abbreviations* SE, standard error; BMI, body mass index; PIR, poverty income ratio

**Fig. 2:**
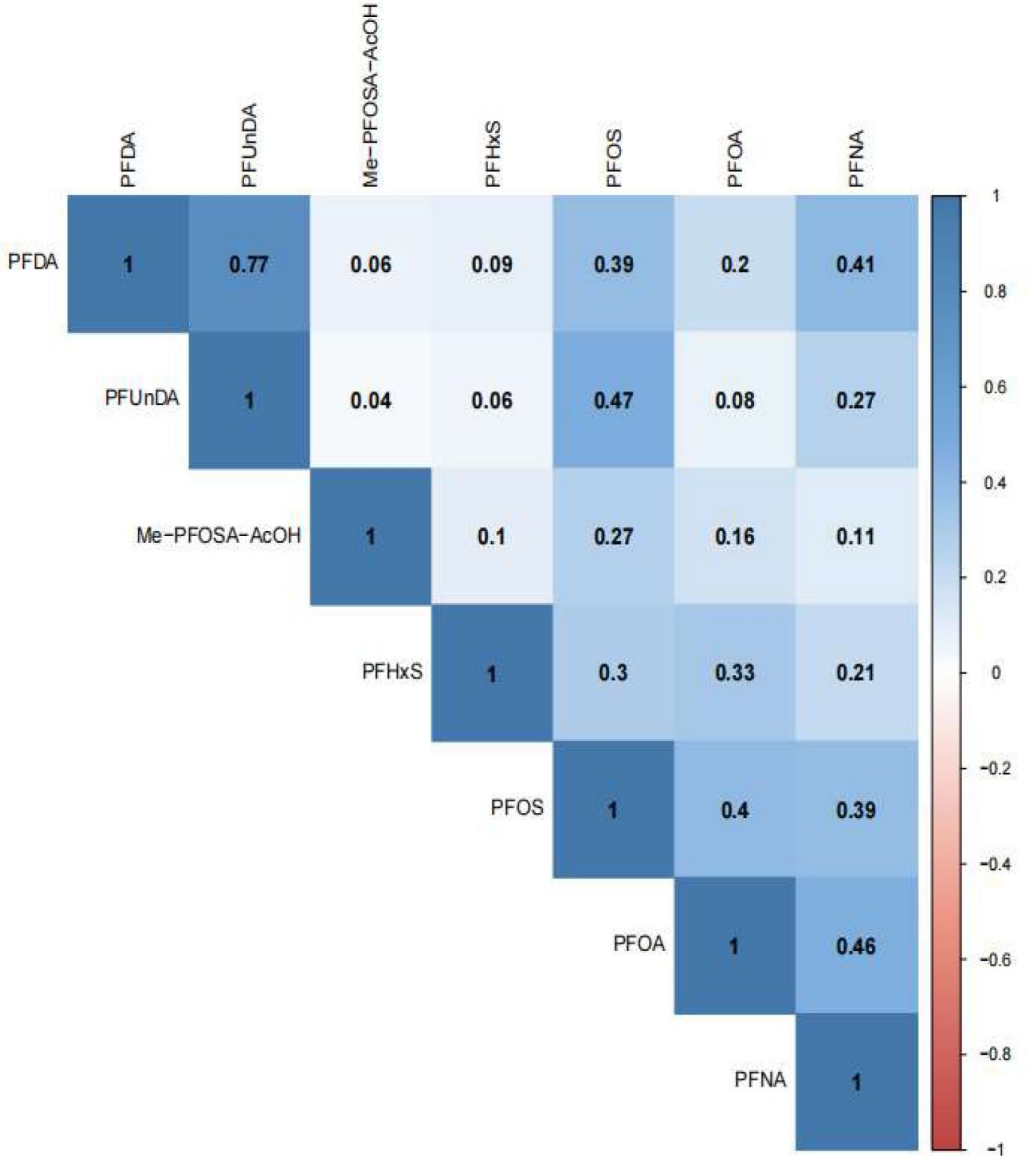
Pearson correlation between serum per- and polyfluoroalkyl substances (PFAS) after Iog_2_ transformed

Table 2 presents the serum concentration distributions and detection frequencies of the seven PFAS analyzed in our study. PFUnDA, Me-PFOSA-AcOH, and PFDA were detected in 46.70%, 57.01%, and 74.91% of participant samples, respectively, while the detection rates for the remaining PFAS exceeded 98%. PFOS exhibited the highest median concentration at 9.70 ng/mL, followed by PFOA at 2.63 ng/mL. The strongest correlation was observed between PFDA and PFUnDA (*r* = 0.77, *P* < 0.001). Correlation coefficients among other PFAS pairs varied, ranging from 0.04 (between PFUnDA and Me-PFOSA-AcOH) to 0.47 (between PFOS and PFUnDA) (Fig. 2).

**Table 2:** Distribution of serum per- and polyfluoroalkyl substance (PFAS) among participants, NHANES 2003–2018 (*N* = 10,496)

PFAS (ng/mL)	Detection frequency (%)	LOD	GM	Mean	Percentile				
					5th	25th	50th	75th	95th
PFOA	99.50	0.1	2.62	3.47	0.67	1.57	2.63	4.30	8.00
PFOS	99.63	0.2	8.90	13.75	1.70	4.90	9.70	18.00	40.40
PFHxS	98.44	0.1	1.50	2.27	0.30	0.82	1.50	2.60	6.30
PFDA	74.91	0.2	0.24	0.34	0.07	0.14	0.20	0.40	1.00
PFNA	98.42	0.1	0.85	1.15	0.20	0.57	0.90	1.40	2.95
PFUnDA	46.70	0.2	0.16	0.23	0.07	0.07	0.14	0.20	0.70
Me-PFOSA-AcOH	57.01	0.2	0.20	0.33	0.06	0.07	0.20	0.37	1.06

*Abbreviations* GM, geometric mean; PFOA, perfluorooctanoic acid; PFOS, perfluorooctane sulfonate; PFHxS, perfluorohexane sulfonic acid; PFDA, perfluorodecanoic acid; PFNA, perfluorononanoic acid; PFUnDA, perfluoroundecanoic acid; Me-PFOSA-AcOH, methyl perfluorooctane sulfonamidoacetic acid

### Associations of individual PFAS with RA

The study predominantly found no significant association between the doubling of PFAS concentrations and the risk of RA, with occasional inverse associations observed (Table 3). Specifically, a doubling in PFOA concentration was associated with a 12% reduction in RA odds (OR = 0.88, 95% CI: 0.79, 0.98) and similar result was observed for a doubling in PFDA and PFNA concentration (OR = 0.89, 95% CI: 0.81, 0.99 and OR = 0.87, 95% CI: 0.77, 0.98, respectively). PFUnDA also exhibited associations with lower RA odds in the partially adjusted model, although these associations were not statistically significant in the fully adjusted models. Conversely, Me-PFOSA-AcOH was linked to increased RA odds in the unadjusted model (OR = 1.13, 95%CI: 1.05, 1.22). The estimates for other PFAS compounds generally showed a consistent direction of effect, but were not statistically significant.

**Table 3:** Associations between single serum per- and polyfluoroalkyl substance (PFAS) and odds of rheumatoid arthritis (RA) in the NHANES 2003–2018 cycles

PFAS (ng/mL)	Unadjusted			Model 1			Model 2		
	OR (95% CI)	*p*-value	*q*-value	OR (95% CI)	*p*-value	*q*-value	OR (95% CI)	*p*-value	*q*-value
**PFOA**									
Per doubling	0.90 (0.81, 1.00)	**0.04**	0.19	0.84 (0.76, 0.94)	**0.002**	**0.03**	0.88 (0.79, 0.98)	**0.02**	0.15
Q1	Ref	–		Ref	–		Ref	–	
Q2	0.72 (0.51, 1.01)	0.06	0.21	0.61 (0.43, 0.88)	**0.01**	**0.03**	0.67 (0.46, 0.98)	**0.04**	0.15
Q3	0.97 (0.70, 1.34)	0.85	0.85	0.78 (0.56, 1.08)	0.13	0.20	0.87 (0.62, 1.22)	0.42	0.47
Q4	0.66 (0.47, 0.91)	**0.01**	0.07	0.54 (0.38, 0.76)	**< 0.001**	**0.03**	0.60 (0.42, 0.86)	**0.01**	0.15
**PFOS**									
Per doubling	1.03 (0.95, 1.13)	0.46	0.56	0.96 (0.88, 1.05)	0.37	0.40	0.97 (0.88, 1.06)	0.45	0.48
Q1	Ref	–		Ref	–		Ref	–	
Q2	0.89 (0.63, 1.25)	0.50	0.58	0.79 (0.56, 1.12)	0.18	0.23	0.82 (0.58, 1.17)	0.27	0.36
Q3	1.07 (0.76, 1.52)	0.68	0.71	0.86 (0.60, 1.23)	0.41	0.43	0.91 (0.64, 1.31)	0.61	0.63
Q4	1.08 (0.76, 1.53)	0.66	0.71	0.81 (0.55, 1.19)	0.29	0.32	0.82 (0.56, 1.22)	0.34	0.41
**PFHxS**									
Per doubling	0.94 (0.86, 1.03)	0.15	0.30	0.88 (0.80, 0.97)	**0.01**	**0.03**	0.91 (0.83, 1.00)	**0.05**	0.15
Q1	Ref	–		Ref	–		Ref	–	
Q2	0.87 (0.64, 1.17)	0.35	0.45	0.73 (0.55, 0.99)	**0.04**	0.07	0.78 (0.57, 1.07)	0.12	0.22
Q3	0.80 (0.58, 1.11)	0.18	0.32	0.64 (0.45, 0.91)	**0.01**	**0.03**	0.70 (0.49, 1.01)	0.06	0.15
Q4	0.80 (0.57, 1.13)	0.21	0.32	0.63 (0.44, 0.90)	**0.01**	**0.03**	0.70 (0.48, 1.00)	0.06	0.15
**PFDA**									
Per doubling	0.90 (0.82, 1.00)	**0.05**	0.20	0.87 (0.78, 0.96)	**0.01**	**0.03**	0.89 (0.81, 0.99)	**0.03**	0.15
Q1	Ref	–		Ref	–		Ref	–	
Q2	0.93 (0.70, 1.24)	0.63	0.71	0.89 (0.66, 1.20)	0.44	0.44	0.97 (0.71, 1.31)	0.82	0.82
Q3	0.77 (0.56, 1.05)	0.10	0.23	0.73 (0.53, 0.99)	**0.04**	0.07	0.81 (0.60, 1.11)	0.19	0.29
Q4	0.76 (0.56, 1.03)	0.08	0.23	0.67 (0.49, 0.92)	**0.01**	**0.03**	0.72 (0.52, 1.00)	**0.05**	0.15
**PFNA**									
Per doubling	0.91 (0.81, 1.02)	0.09	0.23	0.85 (0.76, 0.96)	**0.01**	**0.03**	0.87 (0.77, 0.98)	**0.02**	0.15
Q1	Ref	–		Ref	–		Ref	–	
Q2	0.85 (0.61, 1.17)	0.32	0.43	0.78 (0.56, 1.09)	0.14	0.21	0.82 (0.58, 1.15)	0.25	0.35
Q3	0.76 (0.56, 1.04)	0.09	0.23	0.67 (0.49, 0.92)	**0.01**	**0.03**	0.70 (0.51, 0.96)	**0.03**	0.15
Q4	0.81 (0.58, 1.13)	0.22	0.32	0.70 (0.49, 0.99)	**0.04**	0.07	0.74 (0.51, 1.06)	0.10	0.20
**PFUnDA**									
Per doubling	0.94 (0.85, 1.04)	0.22	0.32	0.89 (0.80, 0.98)	**0.02**	**0.05**	0.91 (0.82, 1.01)	0.07	0.15
Q1	Ref	–		Ref	–		Ref	–	
Q2	0.83 (0.60, 1.14)	0.24	0.34	0.79 (0.58, 1.09)	0.15	0.21	0.86 (0.63, 1.19)	0.36	0.42
Q3	0.83 (0.62, 1.11)	0.22	0.32	0.73 (0.55, 0.98)	**0.04**	0.07	0.81 (0.60, 1.10)	0.18	0.29
Q4	0.77 (0.56, 1.06)	0.11	0.24	0.66 (0.48, 0.90)	**0.01**	**0.03**	0.73 (0.53, 1.02)	0.06	0.15
**Me-PFOSA-AcOH**									
Per doubling	1.13 (1.05, 1.22)	**0.002**	0.06	1.06 (0.98, 1.14)	0.17	0.23	1.05 (0.97, 1.14)	0.20	0.29
Q1	Ref	–		Ref	–		Ref	–	
Q2	1.39 (1.02, 1.90)	**0.04**	0.19	1.22 (0.89, 1.67)	0.21	0.26	1.23 (0.89, 1.69)	0.20	0.29
Q3	1.58 (1.12, 2.22)	**0.01**	0.07	1.39 (0.98, 1.96)	0.06	0.10	1.37 (0.97, 1.93)	0.07	0.15
Q4	1.57 (1.10, 2.23)	**0.01**	0.07	1.21 (0.85, 1.72)	0.29	0.32	1.20 (0.84, 1.72)	0.30	0.38

Me-PFOSA-AcOH, methyl perfluorooctane sulfonamidoacetic acid

PFAS concentration were log_2_-transformed

Model 1 was adjusted for age and sex

Model 2 was adjusted for age, sex, ethnicity, education attainment, body mass index, poverty income ratio, alcohol consumption, physical activity

*q*-value, the False discovery Rate correction (FDR) was implemented

Bold values indicate statistical significance (*p* < 0.05)

*Abbreviations* OR, odds ratio; CI, confidence interval; Q, quartile; PFOA, perfluorooctanoic acid; PFOS, perfluorooctane sulfonate; PFHxS, perfluorohexane sulfonic acid; PFDA, perfluorodecanoic acid; PFNA, perfluorononanoic acid; PFUnDA, perfluoroundecanoic acid

When PFAS levels were categorized into quartiles, similar patterns emerged. For instance, the highest quartile of PFDA exposure was associated with lower RA odds [Q4 vs. Q1: OR = 0.72 (95% CI: 0.52, 1.00)]. PFOA showed a similar association with lower RA odds, with the relationship not being monotonic [Q2 vs. Q1: OR = 0.67 (95% CI: 0.46, 0.98); Q3 vs. Q1: OR = 0.87 (95% CI: 0.62, 1.22); Q4 vs. Q1: OR = 0.60 (95% CI: 0.42, 0.86)]. Assessments of other PFAS exposures in quartiles mostly yielded null results, except for Me-PFOSA-AcOH, which was associated with increased odds of RA in the unadjusted model. After correcting for multiple comparisons in the main analyses, significant p-values from the primary analysis only remained in small number of the analyses.

In subgroup analyses stratified by general characteristics of participants for each PFAS, we found that PFOA, PFDA, PFUnDA, and PFHxS was associated with lower odds of RA in female for both continuous and categorical exposure, and most significantly associations were only existed in some subgroups (Supplemental Table S1–S7). For the remained PFAS, we also found that there are some significant associations in some subgroups for specific exposure pattern.

### Associations of the PFAS mixture with RA

**Fig. 3:**
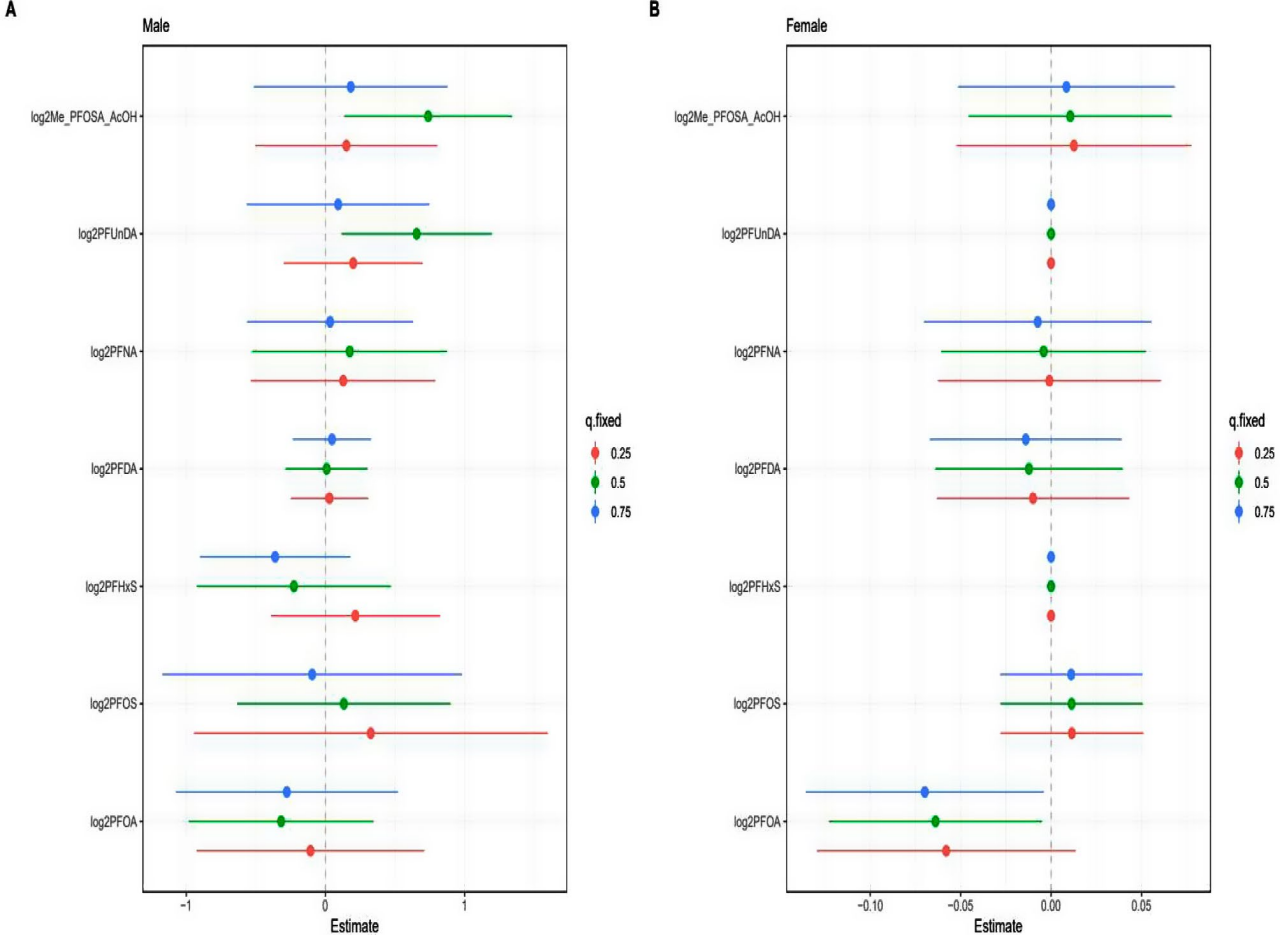
Associations of each individual PFAS with RA status in BKMR model stratified by sex. This plot describes the estimated RA status associated with a change in each individual PFAS from its 25th to 75th percentile, when all the other PFAS are fixed at either the 25th (red line), 50th (green line), or 75th percentile (blue line). Dots indicate the estimate, and horizontal lines indicate the 95% credible intervals. All models were adjusted for age, sex, ethnicity, educational attainment, body mass index, poverty income ratio, alcohol consumption and physical activity

Univariate exposure-response functions for seven PFAS in relation to RA risk, showing no apparent increasing or decreasing trends (Supplemental Fig. S2). BKMR analysis for the overall effect of the PFAS mixture on RA odds indicates that there is not significant association between serum PFAS mixture concentrations and RA odds (Supplemental Fig. S3). When examining the associations between individual PFAS and RA while controlling for other PFAS at the 25th, 50th, and 75th percentiles, no significant associations were found (Supplemental Fig. S4). The PIPs for each PFAS exposure are specified as follows: PFOA, 0.444; PFOS, 0.298; PFHxS, 0.368; PFDA, 0.058; PFNA, 0.052; PFUnDA, 0.242, and Me-PFOSA-AcOH, 0.276. BKMR analyses stratified by sex showed similar results (Supplemental Figs. S5 and S6). However, significant associations of Me-PFOSA-AcOH and PFUnDA with RA were found while controlling for other PFAS at the 50th percentile among males (Fig. 3). In addition, PFOA was found to be associated with lower odds of RA while controlling for other PFAS at the 50th and 75th percentiles among females (Fig. 3).

**Fig. 4:**
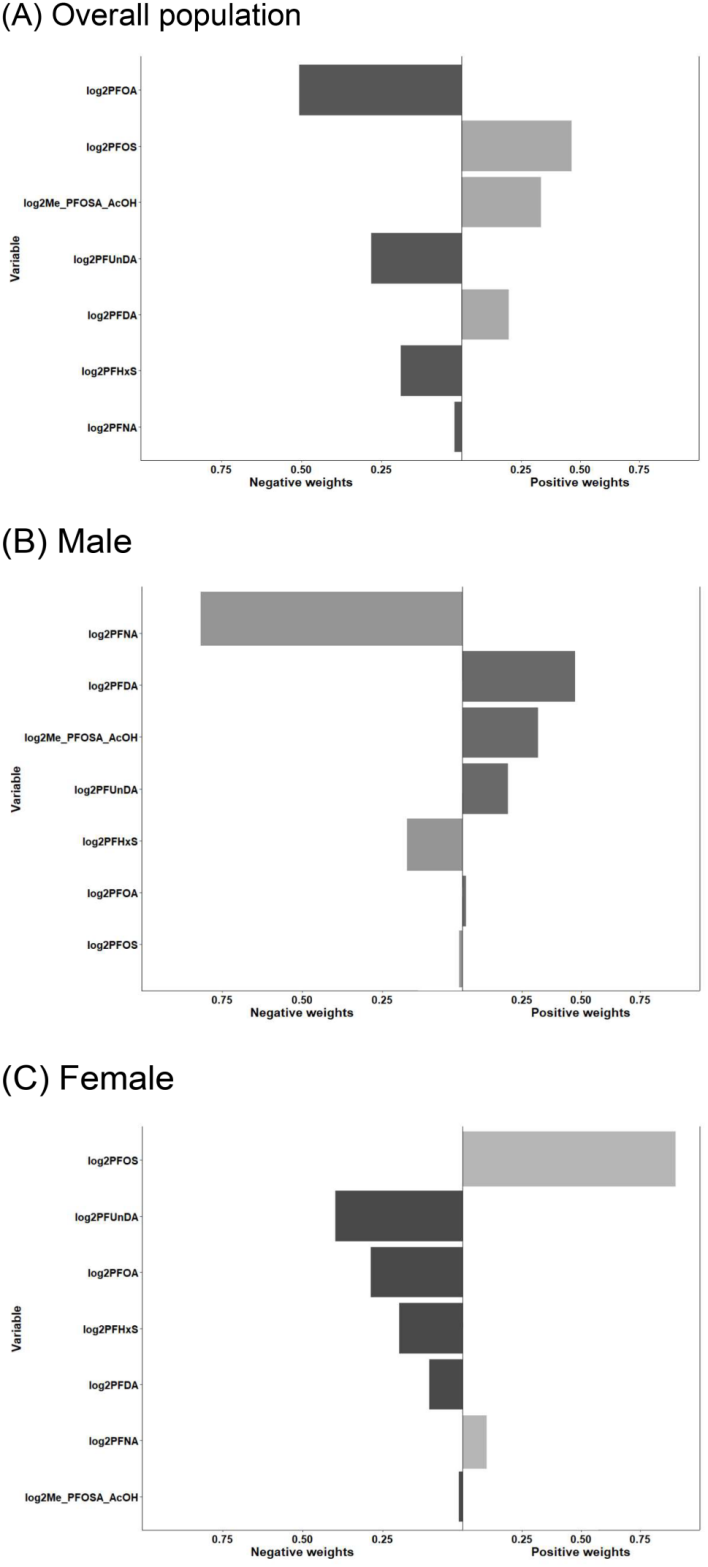
The directions and magnitude of the assigned weights for each log_2_-transformed PFAS in relation to RA status in quantile g-computation for (**A**) overall population, (**B**) male, and (**C**) female. Each weight represents the proportion of the positive or negative partial impact per individual PFAS. The length of each bars indicates the effect size of each exposure in the same direction. All models were adjusted for age, sex, ethnicity, educational attainment, BMI, poverty income ratio, alcohol consumption and physical activity

Quantile g-computation model analysis demonstrated that an increase in PFAS mixture quartile was not linked to higher RA odds (OR = 0.88, 95% CI: 0.76, 1.02). However, a sex-stratified analysis revealed a significant decrease in RA odds among females with a quartile increase in PFAS mixture (OR = 0.76, 95% CI: 0.62, 0.92), but not in males (OR = 1.07, 95% CI: 0.88, 1.29). For females, five serum PFAS were negatively associated with RA risk, while two showed a positive association (Fig. 4). PFOS had the most substantial positive relationship with RA, followed by PFNA. Conversely, PFUnDA led the negative association, followed by PFOA, PFHxS, PFDA, and Me-PFOSA-AcOH.

We also analyzed the chemical mixture’s associations with RA risk in both positive and negative directions using WQS. While the WQS indices in the positive and negative directions were not significantly associated with RA risk. In the fully adjusted models, a quartile increase in the WQS index was linked to a 5% decrease in RA odds (OR = 0.95, 95%CI: 0.89, 1.02) in the negative direction, with PFUnDA contributing the most, followed by PFHxS, PFDA, PFNA and PFOA; however, the effect estimate was not statistically significant. Similar result was also observed in the positive direction (OR = 0.99, 95% CI: 0.94, 1.05), with Me-PFOSA-AcOH contributing the most, followed by PFOS and PFDA. Detailed chemical weight estimates for each WQS index are presented in Supplemental Fig. S7.

Further, sex-specific associations and chemical weight distributions were observed in the repeated hold out WQS analysis with an interaction term (Supplemental Fig. S8A, B). The WQS*sex interaction term was significant (mean OR = 0.91, 95%CI: 0.84, 0.96), with differing slopes for males and females. Females showed a reduced odds of RA (mean OR = 0.93, 95%CI: 0.88, 0.98). Conversely, males demonstrated a marginally increased odds of RA (mean OR = 1.03, 95%CI: 0.98, 1.10); however, this increase was not statistically significant. The distribution of the ORs was opposite for males and females, with 75 out of 100 betas positive for males and 100 out of 100 negative for females. However, significance was only reached for females. These findings are elaborated in Table 4. Notable chemicals of concern differed by sex, with PFUnDA, PFOA, PFHxS, and PFDA for females and PFNA and PFHxS for males (Supplemental Fig. S8B).

**Table 4:** Mean adjusted associations from WQS logistic regression model with 100 repeated holdouts between per- and polyfluoroalkyl substance (PFAS) mixture and odds of rheumatoid arthritis (RA)

RA^a^	Mean OR and SD-based 95% CI^b^	OR < 1^c^
WQS (b_1_)	1.03 (0.98, 1.10)	25/100
WQS*sex (b_12_)	0.91 (0.84, 0.96)	100/100
**OR for male and female**		
Male (b_1_)^d^	1.03 (0.98, 1.10)	25/100
Female (b_2_)^d^	0.93 (0.88, 0.98)	100/100

*Abbreviations* WQS, weighted quantile sum; SD, standard deviation; CI, confidence interval; OR, odds ratio

*Notes* All chemicals were log_2_ transformed to reduce skewness in the distribution of the concentrations

^a^ The associations are derived from a stratified WQS logistic regression model allowing for sex-specific weights and including an interaction term WQS*sex. The model was run with 100 repeated holdouts using 40% of the data as training and 60% as validation set. The models were adjusted for age, sex, ethnicity, educational attainment, BMI, poverty income ratio, alcohol consumption, and physical activity

^b^ The mean OR over the 100 repeated holdouts is presented with its 95% confidence interval

^c^ The number of adjusted ORs from the 100 repeated holdouts that were below 1

^d^ Adjusted OR for male (b_1_) is the OR for the WQS index for the reference group (male = 0), and the adjusted OR for female (b_2_), which is the comparison group (female = 1) is calculated based on the sum of the OR for the WQS*sex interaction term and the OR for the WQS index for the reference group (b_2_ = b_1_ + b_12_). The ORs are calculated based on exponentiation of betas

### Sensitivity analysis

Sensitivity analyses was performed by additionally adjusting the NHANES calendar cycle. There were significantly associations of PFAS exposure with lower odds of RA for both continuous [PFOA: OR = 0.85 (95% CI: 0.76, 0.95); PFHxS: OR = 0.90 (95% CI: 0.82, 0.99); PFDA: OR = 0.88 (95% CI: 0.79, 0.99); PFNA: OR = 0.86 (95% CI: 0.76, 0.97); PFUnDA: OR = 0.88 (95% CI: 0.80, 0.97)] and categorical exposure [PFOA, Q4 vs. Q1: OR = 0.52 (95% CI: 0.37, 0.75); PFHxS, Q4 vs. Q1: OR = 0.68 (95% CI: 0.48, 0.98); PFDA, Q4 vs. Q1: OR = 0.70 (95% CI: 0.50, 0.97); PFUnDA, Q4 vs. Q1: OR = 0.68 (95% CI: 0.48, 0.94)] (Supplemental Table S8). Additionally, the multi-cycle sensitivity analysis revealed significant associations with RA odds for PFOA, PFOS, PFHxS, PFNA, and me-PFOSA-AcOH in at least one cycle (Supplemental Table S9).

## Discussion

### Summary of main results

To the best of our knowledge, this is the first cross-sectional study to assess the association between serum PFAS concentration and RA. In this extensive cross-sectional analysis, we observed a RA prevalence of 5.4%, which is higher than the 0.5 to 1% typically reported in prior research [57, 58]. The discrepancy could be attributed to the exclusion of certain data in our study, implemented to ensure a complete and thorough data analysis. We also identified significant inverse associations of serum PFOA, PFHxS, PFDA, and PFNA levels with the odds of RA. Further, sex-specific analyses within the individual pollutant models indicated that these associations were more marked among females, whereas they were not observed in males. In addition, a stratified analysis of the quantile g-computation by sex revealed a significant association in females. Notably, PFOS demonstrated the most substantial positive influence, whereas PFUnDA showed the most significant negative impact. In the case of WQS regression, we identified a significant WQS*sex interaction term, indicating the presence of sex-specific differences. The results suggest that PFAS, when considered as a mixture, is associated with a reduced odds of RA in females. PFUnDA, PFOA, PFHxS, and PFDA emerged as the primary contributors to this mixed effect. Conversely, WQS analysis did not reveal significant association of the PFAS mixture with RA in males. The BKMR analysis suggests that PFUnDA and Me-PFOSA-AcOH were associated with increased risk of RA in males, while PFOA was associated with lower odds of RA in females.

### Comparison with previous studies and potential biological mechanisms

To our knowledge, only three prior epidemiological studies [31–33] have explored the relationship between PFAS exposure and immune markers relating to RA, disease activity of RA and risk of RA, respectively. One study in China by Zhao et al. [31] analyzed serum from 280 healthy individuals and 294 RA patients, finding several PFAS were associated with elevated immune-related parameters, such as C-reactive protein and IgA. They further reported that PFAS may prompt the disease activity of RA [32]. Qu et al. [33] investigated the association of PFAS with RA in a case-control study, and identified a significant association between PFOA exposure and increased risk of RA [33]. However, these three studies were based on case-control design with small sample size. Our study, using the latest NHANES data from 2003 to 2018, presents a contrasting view. We noted inverse associations of various PFAS, particularly PFOA, PFHxS, PFDA and PFNA, and PFAS mixture with RA in females. These differences may stem from variations in study design, exposure levels, or demographic characteristics. Additionally, our study accounted for numerous covariates such as physical activity and alcohol consumption, which previous studies overlooked.

Despite the scarcity of research on PFAS exposure and RA, there is evidence linking PFAS to suppressed antibody responses after vaccination [59–61], increased risk of infectious diseases [62–65] and allergic outcomes [24], though with both positive and negative associations. Our research identified a significant trend: both individual and mixed PFAS exposures were inversely associated with the odds of RA. However, the cross-sectional nature of our study necessitates a cautious interpretation of these associations. At this stage, it is premature to conclude that PFAS exposure acts as a protective factor against RA. The potential immunosuppressive mechanisms of PFAS, as reviewed in previous literature [66], may explain these findings. In detail, RA is a chronic inflammatory disease characterized by significant immune activation within the synovial compartment of joints and numerous systemic effects [67]. The pathophysiology of RA, although not fully understood, is marked by the dominance of T cells and T cell-derived cytokines in the synovial membrane’s mononuclear infiltrates, highlighting their critical role in the disease’s autoimmune response [68]. Furthermore, the observed upregulation of the Th1 response within the synovial compartment suggests that a Th1/Th2 imbalance is crucial in the pathogenesis of RA [69]. Epidemiological studies have demonstrated a PFAS-induced disruption in this balance, characterized by increased Th2 [70] and decreased Th1 cytokine production [59, 70]. This shift towards a Th2-dominant response has been corroborated in both in vitro [71] and in vivo studies [72, 73]. Additionally, He et al. (2017) found that RA patients typically exhibit a Th1/Th2 balance skewed towards Th1 [74]. These evidence, taken together with our findings, suggests that PFAS exposure may mitigate RA risk by inducing an opposing effect on the Th1/Th2 balance. In summary, mechanistic studies indicate that PFAS exposure can influence Th1/Th2 balance and downstream signaling. However, the mechanism likely extends beyond Th1/Th2 imbalance, possibly involving more specific immune processes, such as calcium signaling, and broader systemic mechanisms like lipid metabolism and oxidative stress, contributing to the immunotoxicity of PFAS [20].

In our study, we specifically investigated how sex modifies the relationship between PFAS exposure and RA. Our findings revealed a striking sex-specific disparity: while PFAS exposure was inversely associated with RA in females, such an association was not evident in males [64, 75]. The literature frequently reports sex-specific differences in the health effects of PFAS [36, 76, 77], but the precise mechanisms underlying these differences remain elusive. One possible factor is the observed variance in PFAS blood concentrations between men and women [78]. Additionally, interactions between PFAS and sex hormones [79–81], particularly estradiol, may play a significant role, as available data support hypoandrogenicity in RA patients [73, 82]. Additionally, sex-specific differences in metabolic and detoxification processes could also modulate the effects of PFAS exposure [83, 84]. To comprehensively understand these contradictory associations, detailed mechanistic studies focusing on individual PFAS chemicals are essential.

### Strengths and limitations

Our study demonstrates several strengths. Firstly, it capitalizes on data from the NHANES, noted for its representative sampling of the U.S. general population. NHANES employs rigorous and standardized methods for data collection via questionnaires and biological sample analysis, also providing sample weights. These methodologies significantly bolster the robustness and reliability of our findings. Secondly, the innovative use of a repeated holdout WQS regression model in our study enhances the stability of the WQS estimates. This model is particularly adept at identifying chemicals of concern and observing sex-specific effects, which are critical aspects of our research.

However, certain limitations warrant consideration. The cross-sectional nature of our study, inherent in its observational design, provides foundational insights into potential associations but falls short in definitively establishing temporal sequences or causality. This necessitates the replication of our results and encourages future longitudinal studies for a more robust validation of the observed associations. Additionally, the reliance on a single time points serum sample assessment to infer long-term PFAS exposure status could potentially limit its scope. Given that one measurement of PFAS in blood is indicative of cumulative exposure spanning 5–10 years [85], and considering the prolonged development period of RA, this approach may overlook crucial long-term chemical influences, thereby introducing potential confounding variables. This study conducted numerous subgroup analyses to uncover associations within particular subgroups. While we did identify some significant associations in certain subgroups, it is important to note the potential risk of false positives arising from multiple comparisons. Therefore, these findings should be interpreted with caution. Finally, the exclusive inclusion of adult participants from the United States in this study constrains the generalizability of our conclusions to other geographic contexts.

## Conclusions

In conclusion, our findings indicate potential inverse associations at background exposure levels between several prominent PFAS and RA risk. It is imperative to conduct further longitudinal studies to explore the effects of PFAS and to elucidate the mechanisms by which PFAS exposure might influence the development of autoimmune diseases. Such research is crucial to both substantiate and elucidate the implications of the findings presented in this study.

### Electronic supplementary material

Below is the link to the electronic supplementary material.

Supplementary Material 1

## Data Availability

All data are open access and available for download at url: https://www.cdc.gov/nchs/nhanes/index.htm.
