# Supplementary material for: Associations of per- and polyfluoroalkyl substances (PFAS) and their mixture with risk of rheumatoid arthritis in the U.S. adult population
Source: Environ Health. 2024 Apr 13;23:38. doi: 10.1186/s12940-024-01073-3 (PMC11015572; doi:10.1186/s12940-024-01073-3)
Supplement: Supplementary file 1 — Supplementary Material 1 [file 12940_2024_1073_MOESM1_ESM.docx]

**Supplemental Table S1.** Subgroup analysis of the association between PFOA exposure and odds of RA by the general characteristics of participants in fully adjusted model.

| **Characteristic** | **Continuous** | | **Q1** | **Q2** | | **Q3** | | **Q4** | |
| --- | --- | --- | --- | --- | --- | --- | --- | --- | --- |
|  | **OR (95% CI)** | ***p*** |  | **OR (95% CI)** | ***p*** | **OR (95% CI)** | ***p*** | **OR (95% CI)** | ***p*** |
| **Age** |  |  |  |  |  |  |  |  |  |
| 20 – 39 | 0.95 (0.74, 1.23) | 0.72 | Ref | 1.75 (0.58, 5.30) | 0.32 | 1.17 (0.40, 3.39) | 0.77 | 0.67 (0.22, 2.02) | 0.47 |
| 40 – 59 | 0.90 (0.74, 1.10) | 0.29 | Ref | 0.50 (0.29, 0.88) | **0.02** | 0.80 (0.46, 1.40) | 0.43 | 0.52 (0.27, 1.01) | **0.05** |
| ≥ 60 | 0.86 (0.73, 1.02) | 0.08 | Ref | 0.69 (0.43, 1.10) | 0.12 | 0.89 (0.56, 1.44) | 0.64 | 0.70 (0.44, 1.10) | 0.12 |
| **Sex** |  |  |  |  |  |  |  |  |  |
| Male | 1.00 (0.83, 1.20) | 1 | Ref | 0.72 (0.37, 1.40) | 0.33 | 0.80 (0.45, 1.41) | 0.43 | 0.78 (0.45, 1.35) | 0.37 |
| Female | 0.82 (0.71, 0.95) | **0.01** | Ref | 0.65 (0.42, 0.99) | **0.05** | 0.92 (0.59, 1.45) | 0.73 | 0.47 (0.28, 0.79) | **0.005** |
| **Ethnicity** |  |  |  |  |  |  |  |  |  |
| Non-Hispanic White | 0.87 (0.75, 1.01) | 0.06 | Ref | 0.60 (0.35, 1.03) | 0.06 | 0.80 (0.50, 1.29) | 0.35 | 0.58 (0.37, 0.91) | **0.02** |
| Non-Hispanic Black | 0.96 (0.82, 1.13) | 0.63 | Ref | 1.07 (0.64, 1.80) | 0.79 | 0.82 (0.45, 1.50) | 0.53 | 0.94 (0.57, 1.56) | 0.8 |
| Mexican American | 0.75 (0.60, 0.96) | **0.02** | Ref | 0.73 (0.33, 1.62) | 0.44 | 0.77 (0.36, 1.67) | 0.51 | 0.19 (0.06, 0.60) | **0.01** |
| Other | 0.87 (0.70, 1.08) | 0.2 | Ref | 0.51 (0.24, 1.10) | 0.09 | 1.51 (0.72, 3.16) | 0.27 | 0.14 (0.03, 0.62) | **0.01** |
| **Poverty income ratio** |  |  |  |  |  |  |  |  |  |
| ≤1.30 | 1.01 (0.87, 1.17) | 0.94 | Ref | 0.84 (0.53, 1.33) | 0.45 | 1.37 (0.81, 2.31) | 0.23 | 0.77 (0.47, 1.26) | 0.3 |
| 1.31 – 3.50 | 0.89 (0.76, 1.04) | 0.13 | Ref | 0.80 (0.45, 1.43) | 0.44 | 0.88 (0.52, 1.50) | 0.65 | 0.59 (0.34, 1.04) | 0.07 |
| >3.5 | 0.78 (0.62, 0.98) | **0.03** | Ref | 0.42 (0.20, 0.87) | **0.02** | 0.55 (0.27, 1.10) | 0.09 | 0.48 (0.25, 0.94) | **0.03** |
| **Educational attainment** |  |  |  |  |  |  |  |  |  |
| <High school | 0.85 (0.71, 1.02) | 0.09 | Ref | 0.55 (0.32, 0.97) | **0.04** | 0.67 (0.36, 1.26) | 0.21 | 0.63 (0.36, 1.13) | 0.12 |
| ≥High school | 0.89 (0.79, 1.01) | 0.06 | Ref | 0.70 (0.46, 1.09) | 0.11 | 0.92 (0.63, 1.35) | 0.68 | 0.58 (0.37, 0.89) | **0.01** |
| **BMI** |  |  |  |  |  |  |  |  |  |
| 25-30.0 | 1.02 (0.85, 1.21) | 0.86 | Ref | 0.71 (0.34, 1.45) | 0.34 | 1.24 (0.65, 2.38) | 0.51 | 1.04 (0.56, 1.95) | 0.9 |
| <25 | 0.87 (0.70, 1.09) | 0.22 | Ref | 0.61 (0.31, 1.21) | 0.16 | 0.61 (0.29, 1.31) | 0.21 | 0.54 (0.24, 1.21) | 0.13 |
| ≥30.0 | 0.82 (0.71, 0.95) | **0.01** | Ref | 0.70 (0.42, 1.18) | 0.18 | 0.84 (0.53, 1.35) | 0.48 | 0.42 (0.26, 0.67) | **<0.001** |
| **Alcohol consumption** |  |  |  |  |  |  |  |  |  |
| Never | 0.85 (0.69, 1.05) | 0.13 | Ref | 0.75 (0.34, 1.67) | 0.48 | 1.09 (0.53, 2.25) | 0.81 | 0.53 (0.25, 1.15) | 0.11 |
| Former | 0.81 (0.67, 0.99) | **0.04** | Ref | 0.50 (0.26, 0.99) | **0.05** | 0.54 (0.27, 1.11) | 0.09 | 0.50 (0.24, 1.04) | 0.06 |
| Mild | 0.88 (0.73, 1.06) | 0.16 | Ref | 0.72 (0.35, 1.45) | 0.35 | 0.97 (0.53, 1.76) | 0.92 | 0.56 (0.31, 1.00) | **0.05** |
| Moderate | 1.11 (0.84, 1.47) | 0.47 | Ref | 1.09 (0.40, 2.94) | 0.86 | 1.98 (0.72, 5.43) | 0.18 | 1.15 (0.41, 3.22) | 0.79 |
| Heavy | 0.96 (0.68, 1.37) | 0.84 | Ref | 0.61 (0.23, 1.62) | 0.32 | 0.66 (0.24, 1.77) | 0.4 | 0.71 (0.25, 2.05) | 0.52 |
| **Physical activity** |  |  |  |  |  |  |  |  |  |
| No | 0.91 (0.79, 1.04) | 0.16 | Ref | 0.70 (0.45, 1.09) | 0.11 | 0.98 (0.61, 1.58) | 0.94 | 0.63 (0.40, 0.99) | **0.04** |
| Yes | 0.84 (0.71, 1.00) | **0.04** | Ref | 0.62 (0.34, 1.13) | 0.12 | 0.72 (0.44, 1.17) | 0.19 | 0.56 (0.35, 0.92) | **0.02** |

Notes: Fully adjusted model, adjusted for age, sex, ethnicity, educational attainment, BMI, poverty income ratio, alcohol consumption and physical activity. Continuous, per doubling concentration of PFOA. CI: confidence interval; OR: odds ratio; Q, quartile; Ref, reference. BMI, body mass index.

Bold values indicate statistical significance (*p* < 0.05).

**Supplemental Table S2.** Subgroup analysis of the association between PFOS exposure and odds of RA by the general characteristics of participants in fully adjusted model.

| **Characteristic** | **Continuous** | | **Q1** | **Q2** | | **Q3** | | **Q4** | |
| --- | --- | --- | --- | --- | --- | --- | --- | --- | --- |
|  | **OR (95% CI)** | ***p*** |  | **OR (95% CI)** | ***p*** | **OR (95% CI)** | ***p*** | **OR (95% CI)** | ***p*** |
| **Age** |  |  |  |  |  |  |  |  |  |
| 20 – 39 | 1.04 (0.80, 1.36) | 0.74 | Ref | 1.21 (0.48, 3.10) | 0.68 | 1.61 (0.58, 4.42) | 0.36 | 0.45 (0.08, 2.43) | 0.35 |
| 40 – 59 | 0.95 (0.83, 1.09) | 0.47 | Ref | 0.68 (0.38, 1.22) | 0.2 | 0.68 (0.37, 1.23) | 0.2 | 0.71 (0.36, 1.40) | 0.32 |
| ≥ 60 | 0.99 (0.86, 1.14) | 0.94 | Ref | 1.04 (0.60, 1.83) | 0.88 | 1.31 (0.78, 2.20) | 0.31 | 1.22 (0.72, 2.05) | 0.46 |
| **Sex** |  |  |  |  |  |  |  |  |  |
| Male | 1.07 (0.94, 1.22) | 0.31 | Ref | 0.95 (0.45, 1.99) | 0.89 | 1.05 (0.50, 2.21) | 0.89 | 1.06 (0.50, 2.26) | 0.87 |
| Female | 0.91 (0.81, 1.03) | 0.15 | Ref | 0.78 (0.52, 1.17) | 0.22 | 0.86 (0.55, 1.35) | 0.51 | 0.70 (0.42, 1.16) | 0.16 |
| **Ethnicity** |  |  |  |  |  |  |  |  |  |
| Non-Hispanic White | 0.94 (0.83, 1.05) | 0.27 | Ref | 0.71 (0.43, 1.15) | 0.16 | 0.79 (0.49, 1.28) | 0.34 | 0.76 (0.48, 1.21) | 0.25 |
| Non-Hispanic Black | 1.04 (0.92, 1.18) | 0.5 | Ref | 1.26 (0.77, 2.06) | 0.35 | 1.30 (0.76, 2.23) | 0.34 | 1.04 (0.55, 1.98) | 0.91 |
| Mexican American | 0.90 (0.72, 1.13) | 0.35 | Ref | 0.66 (0.35, 1.25) | 0.2 | 1.30 (0.64, 2.63) | 0.46 | 0.39 (0.16, 0.91) | **0.03** |
| Other | 1.01 (0.84, 1.22) | 0.93 | Ref | 1.14 (0.55, 2.38) | 0.72 | 1.02 (0.45, 2.28) | 0.96 | 0.86 (0.33, 2.22) | 0.75 |
| **Poverty income ratio** |  |  |  |  |  |  |  |  |  |
| ≤ 1.30 | 1.00 (0.87, 1.16) | 0.97 | Ref | 1.15 (0.69, 1.91) | 0.58 | 1.12 (0.64, 1.97) | 0.69 | 0.96 (0.48, 1.93) | 0.91 |
| 1.31 – 3.50 | 0.98 (0.85, 1.12) | 0.73 | Ref | 0.88 (0.49, 1.57) | 0.67 | 0.94 (0.53, 1.67) | 0.84 | 0.94 (0.55, 1.60) | 0.82 |
| > 3.5 | 0.92 (0.77, 1.09) | 0.33 | Ref | 0.56 (0.27, 1.13) | 0.1 | 0.73 (0.37, 1.41) | 0.34 | 0.60 (0.30, 1.21) | 0.15 |
| **Educational attainment** |  |  |  |  |  |  |  |  |  |
| < High school | 0.89 (0.77, 1.02) | 0.08 | Ref | 0.84 (0.48, 1.46) | 0.53 | 0.69 (0.41, 1.16) | 0.16 | 0.57 (0.33, 0.98) | **0.04** |
| ≥ High school | 0.99 (0.89, 1.10) | 0.86 | Ref | 0.82 (0.54, 1.25) | 0.35 | 0.98 (0.64, 1.49) | 0.92 | 0.90 (0.57, 1.41) | 0.63 |
| **BMI** |  |  |  |  |  |  |  |  |  |
| 25 – 30.0 | 1.04 (0.89, 1.20) | 0.63 | Ref | 0.84 (0.41, 1.70) | 0.62 | 1.04 (0.54, 2.01) | 0.91 | 1.05 (0.54, 2.05) | 0.88 |
| < 25 | 0.96 (0.81, 1.13) | 0.63 | Ref | 0.59 (0.26, 1.32) | 0.19 | 0.59 (0.28, 1.24) | 0.16 | 0.70 (0.32, 1.52) | 0.36 |
| ≥ 30.0 | 0.92 (0.81, 1.05) | 0.23 | Ref | 0.98 (0.63, 1.51) | 0.93 | 1.06 (0.65, 1.74) | 0.81 | 0.74 (0.45, 1.21) | 0.22 |
| **Alcohol consumption** |  |  |  |  |  |  |  |  |  |
| Never | 0.87 (0.74, 1.02) | 0.09 | Ref | 0.84 (0.39, 1.85) | 0.67 | 1.11 (0.53, 2.32) | 0.77 | 0.57 (0.27, 1.20) | 0.14 |
| Former | 0.95 (0.79, 1.14) | 0.56 | Ref | 0.79 (0.36, 1.76) | 0.56 | 0.91 (0.44, 1.87) | 0.8 | 0.64 (0.30, 1.38) | 0.25 |
| Mild | 1.03 (0.85, 1.24) | 0.75 | Ref | 1.33 (0.72, 2.46) | 0.36 | 1.26 (0.64, 2.49) | 0.5 | 1.37 (0.70, 2.66) | 0.36 |
| Moderate | 0.97 (0.82, 1.13) | 0.67 | Ref | 1.09 (0.42, 2.81) | 0.86 | 0.90 (0.33, 2.49) | 0.84 | 0.68 (0.23, 1.98) | 0.47 |
| Heavy | 1.01 (0.79, 1.31) | 0.92 | Ref | 0.26 (0.11, 0.61) | **0.002** | 0.48 (0.16, 1.40) | 0.17 | 0.88 (0.35, 2.24) | 0.79 |
| **Physical activity** |  |  |  |  |  |  |  |  |  |
| No | 0.98 (0.87, 1.10) | 0.72 | Ref | 0.82 (0.53, 1.27) | 0.38 | 0.85 (0.53, 1.36) | 0.51 | 0.88 (0.53, 1.47) | 0.63 |
| Yes | 0.94 (0.82, 1.08) | 0.4 | Ref | 0.82 (0.44, 1.50) | 0.51 | 0.99 (0.57, 1.70) | 0.96 | 0.74 (0.42, 1.28) | 0.28 |

Notes: Fully adjusted model, adjusted for age, sex, ethnicity, educational attainment, BMI, poverty income ratio, alcohol consumption and physical activity. Continuous, per doubling concentration of PFOS. CI: confidence interval; OR: odds ratio; Q, quartile; Ref, reference. BMI, body mass index.

Bold values indicate statistical significance (*p* < 0.05).

**Supplemental Table S3.** Subgroup analysis of the association between PFHxS exposure and odds of RA by the general characteristics of participants in fully adjusted model.

| **Characteristic** | **Continuous** | | **Q1** | **Q2** | | **Q3** | | **Q4** | |
| --- | --- | --- | --- | --- | --- | --- | --- | --- | --- |
|  | **OR (95% CI)** | ***p*** |  | **OR (95% CI)** | ***p*** | **OR (95% CI)** | ***p*** | **OR (95% CI)** | ***p*** |
| **Age** |  |  |  |  |  |  |  |  |  |
| 20 – 39 | 0.92 (0.73, 1.18) | 0.51 | Ref | 1.34 (0.61, 2.95) | 0.46 | 1.37 (0.48, 3.93) | 0.55 | 0.98 (0.31, 3.13) | 0.97 |
| 40 – 59 | 0.99 (0.85, 1.16) | 0.93 | Ref | 0.60 (0.34, 1.05) | 0.07 | 0.64 (0.37, 1.13) | 0.12 | 0.85 (0.47, 1.55) | 0.6 |
| ≥ 60 | 0.86 (0.74, 1.00) | **0.05** | Ref | 0.87 (0.53, 1.44) | 0.6 | 0.70 (0.44, 1.09) | 0.11 | 0.63 (0.39, 1.01) | 0.06 |
| **Sex** |  |  |  |  |  |  |  |  |  |
| Male | 1.02 (0.90, 1.15) | 0.77 | Ref | 1.27 (0.61, 2.63) | 0.51 | 1.34 (0.69, 2.58) | 0.38 | 1.26 (0.64, 2.51) | 0.5 |
| Female | 0.85 (0.73, 0.98) | **0.03** | Ref | 0.70 (0.49, 1.01) | **0.05** | 0.52 (0.31, 0.87) | **0.01** | 0.54 (0.32, 0.90) | **0.02** |
| **Ethnicity** |  |  |  |  |  |  |  |  |  |
| Non-Hispanic White | 0.89 (0.77, 1.02) | 0.1 | Ref | 0.71 (0.46, 1.08) | 0.11 | 0.56 (0.33, 0.93) | **0.03** | 0.62 (0.38, 1.00) | **0.05** |
| Non-Hispanic Black | 0.94 (0.83, 1.08) | 0.39 | Ref | 1.05 (0.61, 1.81) | 0.87 | 1.20 (0.75, 1.91) | 0.44 | 0.94 (0.54, 1.64) | 0.83 |
| Mexican American | 0.81 (0.63, 1.02) | 0.08 | Ref | 0.60 (0.29, 1.26) | 0.18 | 0.65 (0.29, 1.45) | 0.29 | 0.61 (0.26, 1.45) | 0.26 |
| Other | 0.95 (0.73, 1.24) | 0.72 | Ref | 0.68 (0.29, 1.56) | 0.36 | 1.01 (0.41, 2.50) | 0.99 | 0.76 (0.27, 2.12) | 0.6 |
| **Poverty income ratio** |  |  |  |  |  |  |  |  |  |
| ≤ 1.30 | 1.08 (0.93, 1.24) | 0.32 | Ref | 1.00 (0.62, 1.61) | 1 | 1.25 (0.76, 2.03) | 0.38 | 1.07 (0.60, 1.92) | 0.82 |
| 1.31 – 3.50 | 0.83 (0.73, 0.95) | **0.01** | Ref | 0.72 (0.43, 1.21) | 0.22 | 0.59 (0.33, 1.05) | 0.07 | 0.45 (0.25, 0.83) | **0.01** |
| > 3.5 | 0.87 (0.72, 1.05) | 0.15 | Ref | 0.71 (0.38, 1.34) | 0.29 | 0.51 (0.23, 1.14) | 0.1 | 0.78 (0.44, 1.38) | 0.39 |
| **Educational attainment** |  |  |  |  |  |  |  |  |  |
| < High school | 0.98 (0.83, 1.15) | 0.77 | Ref | 1.10 (0.62, 1.97) | 0.74 | 0.99 (0.57, 1.71) | 0.97 | 1.13 (0.59, 2.15) | 0.71 |
| ≥ High school | 0.89 (0.81, 0.98) | **0.02** | Ref | 0.72 (0.50, 1.05) | 0.08 | 0.65 (0.43, 0.98) | **0.04** | 0.62 (0.42, 0.91) | **0.01** |
| **BMI** |  |  |  |  |  |  |  |  |  |
| 25 – 30.0 | 1.03 (0.89, 1.20) | 0.67 | Ref | 1.00 (0.52, 1.92) | 0.99 | 1.09 (0.55, 2.18) | 0.81 | 1.08 (0.54, 2.14) | 0.84 |
| < 25 | 0.89 (0.73, 1.09) | 0.27 | Ref | 0.59 (0.29, 1.21) | 0.15 | 0.61 (0.29, 1.27) | 0.19 | 0.59 (0.30, 1.17) | 0.13 |
| ≥ 30.0 | 0.84 (0.74, 0.95) | **0.01** | Ref | 0.81 (0.51, 1.30) | 0.37 | 0.58 (0.35, 0.95) | **0.03** | 0.57 (0.35, 0.92) | **0.02** |
| **Alcohol consumption** |  |  |  |  |  |  |  |  |  |
| Never | 0.83 (0.68, 1.01) | 0.06 | Ref | 0.69 (0.38, 1.26) | 0.22 | 0.47 (0.23, 0.97) | **0.04** | 0.50 (0.25, 1.01) | **0.05** |
| Former | 0.87 (0.73, 1.05) | 0.14 | Ref | 1.03 (0.53, 1.99) | 0.93 | 0.88 (0.45, 1.74) | 0.71 | 0.66 (0.32, 1.36) | 0.26 |
| Mild | 0.97 (0.80, 1.16) | 0.72 | Ref | 0.70 (0.36, 1.36) | 0.29 | 0.60 (0.31, 1.16) | 0.13 | 0.72 (0.36, 1.44) | 0.35 |
| Moderate | 0.89 (0.66, 1.20) | 0.44 | Ref | 0.77 (0.31, 1.93) | 0.58 | 0.71 (0.26, 1.91) | 0.5 | 0.86 (0.32, 2.33) | 0.77 |
| Heavy | 0.94 (0.77, 1.16) | 0.58 | Ref | 0.71 (0.31, 1.60) | 0.4 | 0.93 (0.40, 2.15) | 0.86 | 0.79 (0.34, 1.84) | 0.58 |
| **Physical activity** |  |  |  |  |  |  |  |  |  |
| No | 0.90 (0.81, 1.01) | 0.07 | Ref | 0.78 (0.53, 1.15) | 0.21 | 0.79 (0.50, 1.24) | 0.3 | 0.55 (0.35, 0.89) | **0.01** |
| Yes | 0.91 (0.78, 1.06) | 0.23 | Ref | 0.76 (0.44, 1.32) | 0.32 | 0.58 (0.34, 0.98) | **0.04** | 0.90 (0.55, 1.45) | 0.66 |

Notes: Fully adjusted model, adjusted for age, sex, ethnicity, educational attainment, BMI, poverty income ratio, alcohol consumption and physical activity. Continuous, per doubling concentration of PFHxS. CI: confidence interval; OR: odds ratio; Q, quartile; Ref, reference. BMI, body mass index.

Bold values indicate statistical significance (*p* < 0.05).

**Supplemental Table S4.** Subgroup analysis of the association between PFDA exposure and odds of RA by the general characteristics of participants in fully adjusted model.

| **Characteristic** | **Continuous** | | **Q1** | **Q2** | | **Q3** | | **Q4** | |
| --- | --- | --- | --- | --- | --- | --- | --- | --- | --- |
|  | **OR (95% CI)** | ***p*** |  | **OR (95% CI)** | ***p*** | **OR (95% CI)** | ***p*** | **OR (95% CI)** | ***p*** |
| **Age** |  |  |  |  |  |  |  |  |  |
| 20 – 39 | 0.81 (0.56, 1.18) | 0.26 | Ref | 1.34 (0.53, 3.43) | 0.54 | 0.95 (0.31, 2.93) | 0.93 | 0.22 (0.04, 1.18) | 0.08 |
| 40 – 59 | 0.82 (0.70, 0.97) | **0.02** | Ref | 0.95 (0.58, 1.57) | 0.85 | 0.61 (0.37, 0.99) | **0.05** | 0.61 (0.35, 1.08) | 0.09 |
| ≥ 60 | 0.98 (0.86, 1.11) | 0.73 | Ref | 0.94 (0.63, 1.40) | 0.76 | 1.01 (0.64, 1.61) | 0.96 | 0.90 (0.61, 1.35) | 0.62 |
| **Sex** |  |  |  |  |  |  |  |  |  |
| Male | 0.98 (0.83, 1.15) | 0.81 | Ref | 1.10 (0.66, 1.81) | 0.72 | 0.74 (0.45, 1.23) | 0.25 | 0.94 (0.55, 1.59) | 0.8 |
| Female | 0.84 (0.73, 0.96) | **0.01** | Ref | 0.89 (0.61, 1.31) | 0.55 | 0.86 (0.59, 1.26) | 0.43 | 0.59 (0.38, 0.91) | **0.02** |
| **Ethnicity** |  |  |  |  |  |  |  |  |  |
| Non-Hispanic White | 0.88 (0.76, 1.01) | 0.07 | Ref | 1.00 (0.68, 1.48) | 0.99 | 0.85 (0.57, 1.28) | 0.43 | 0.63 (0.38, 1.04) | 0.07 |
| Non-Hispanic Black | 1.01 (0.87, 1.19) | 0.86 | Ref | 1.11 (0.64, 1.92) | 0.71 | 1.02 (0.60, 1.73) | 0.94 | 1.22 (0.70, 2.14) | 0.47 |
| Mexican American | 0.76 (0.56, 1.02) | 0.07 | Ref | 0.99 (0.48, 2.07) | 0.99 | 0.57 (0.27, 1.19) | 0.13 | 0.23 (0.08, 0.62) | **0.005** |
| Other | 0.82 (0.64, 1.04) | 0.1 | Ref | 0.60 (0.27, 1.32) | 0.2 | 0.60 (0.24, 1.53) | 0.28 | 0.58 (0.28, 1.18) | 0.13 |
| **Poverty income ratio** |  |  |  |  |  |  |  |  |  |
| ≤ 1.30 | 0.98 (0.82, 1.18) | 0.85 | Ref | 0.91 (0.54, 1.55) | 0.73 | 0.98 (0.57, 1.68) | 0.94 | 0.86 (0.50, 1.49) | 0.59 |
| 1.31 – 3.50 | 0.86 (0.72, 1.01) | 0.07 | Ref | 0.99 (0.61, 1.62) | 0.97 | 0.84 (0.49, 1.44) | 0.53 | 0.70 (0.40, 1.23) | 0.21 |
| > 3.5 | 0.88 (0.72, 1.07) | 0.21 | Ref | 0.94 (0.48, 1.86) | 0.86 | 0.70 (0.37, 1.30) | 0.25 | 0.66 (0.33, 1.31) | 0.23 |
| **Educational attainment** |  |  |  |  |  |  |  |  |  |
| < High school | 0.87 (0.74, 1.02) | 0.09 | Ref | 0.62 (0.39, 0.99) | **0.05** | 0.78 (0.47, 1.30) | 0.34 | 0.58 (0.34, 0.98) | **0.04** |
| ≥ High school | 0.90 (0.80, 1.02) | 0.1 | Ref | 1.07 (0.73, 1.56) | 0.73 | 0.82 (0.56, 1.21) | 0.32 | 0.77 (0.52, 1.14) | 0.19 |
| **BMI** |  |  |  |  |  |  |  |  |  |
| 25 – 30.0 | 0.97 (0.81, 1.16) | 0.75 | Ref | 1.05 (0.59, 1.90) | 0.86 | 1.16 (0.67, 1.99) | 0.59 | 0.90 (0.46, 1.77) | 0.77 |
| < 25 | 0.84 (0.68, 1.04) | 0.12 | Ref | 0.78 (0.37, 1.64) | 0.51 | 0.62 (0.30, 1.27) | 0.19 | 0.58 (0.30, 1.13) | 0.11 |
| ≥ 30.0 | 0.88 (0.76, 1.01) | 0.07 | Ref | 1.06 (0.72, 1.56) | 0.75 | 0.75 (0.47, 1.21) | 0.24 | 0.72 (0.45, 1.15) | 0.17 |
| **Alcohol consumption** |  |  |  |  |  |  |  |  |  |
| Never | 0.84 (0.68, 1.04) | 0.1 | Ref | 1.24 (0.62, 2.49) | 0.54 | 0.77 (0.39, 1.53) | 0.45 | 0.56 (0.25, 1.24) | 0.15 |
| Former | 0.82 (0.64, 1.06) | 0.12 | Ref | 0.71 (0.40, 1.25) | 0.23 | 0.48 (0.24, 0.95) | **0.04** | 0.67 (0.36, 1.27) | 0.22 |
| Mild | 1.01 (0.87, 1.18) | 0.86 | Ref | 1.31 (0.69, 2.46) | 0.4 | 1.47 (0.82, 2.66) | 0.2 | 0.92 (0.49, 1.75) | 0.8 |
| Moderate | 0.96 (0.71, 1.30) | 0.8 | Ref | 1.42 (0.51, 3.94) | 0.49 | 1.31 (0.45, 3.79) | 0.61 | 0.98 (0.32, 2.98) | 0.97 |
| Heavy | 0.83 (0.60, 1.16) | 0.28 | Ref | 0.70 (0.30, 1.63) | 0.41 | 0.33 (0.12, 0.91) | **0.03** | 0.70 (0.28, 1.77) | 0.45 |
| **Physical activity** |  |  |  |  |  |  |  |  |  |
| No | 0.94 (0.83, 1.06) | 0.29 | Ref | 0.93 (0.62, 1.41) | 0.74 | 0.91 (0.61, 1.36) | 0.64 | 0.81 (0.54, 1.23) | 0.32 |
| Yes | 0.83 (0.69, 0.99) | **0.04** | Ref | 0.98 (0.62, 1.56) | 0.95 | 0.67 (0.40, 1.11) | 0.12 | 0.59 (0.34, 1.01) | 0.06 |

Notes: Fully adjusted model, adjusted for age, sex, ethnicity, educational attainment, BMI, poverty income ratio, alcohol consumption and physical activity. Continuous, per doubling concentration of PFDA. CI: confidence interval; OR: odds ratio; Q, quartile; Ref, reference. BMI, body mass index.

Bold values indicate statistical significance (*p* < 0.05).

**Supplemental Table S5.** Subgroup analysis of the association between PFNA exposure and odds of RA by the general characteristics of participants in fully adjusted model.

| **Characteristic** | **Continuous** | | **Q1** | **Q2** | | **Q3** | | **Q4** | |
| --- | --- | --- | --- | --- | --- | --- | --- | --- | --- |
|  | **OR (95% CI)** | ***p*** |  | **OR (95% CI)** | ***p*** | **OR (95% CI)** | ***p*** | **OR (95% CI)** | ***p*** |
| **Age** |  |  |  |  |  |  |  |  |  |
| 20 – 39 | 0.94 (0.71, 1.24) | 0.65 | Ref | 1.77 (0.74, 4.22) | 0.2 | 1.02 (0.35, 2.95) | 0.98 | 0.86 (0.24, 3.11) | 0.82 |
| 40 – 59 | 0.82 (0.67, 1.01) | 0.06 | Ref | 0.68 (0.38, 1.20) | 0.18 | 0.49 (0.27, 0.87) | **0.02** | 0.71 (0.40, 1.25) | 0.23 |
| ≥ 60 | 0.89 (0.75, 1.05) | 0.17 | Ref | 0.77 (0.49, 1.22) | 0.26 | 0.86 (0.52, 1.40) | 0.53 | 0.71 (0.45, 1.12) | 0.14 |
| **Sex** |  |  |  |  |  |  |  |  |  |
| Male | 0.94 (0.78, 1.12) | 0.48 | Ref | 0.87 (0.48, 1.59) | 0.65 | 0.70 (0.40, 1.23) | 0.21 | 0.85 (0.48, 1.53) | 0.86 |
| Female | 0.83 (0.71, 0.97) | **0.02** | Ref | 0.79 (0.53, 1.20) | 0.27 | 0.70 (0.48, 1.02) | 0.07 | 0.65 (0.40, 1.06) | 0.06 |
| **Ethnicity** |  |  |  |  |  |  |  |  | 0.41 |
| Non-Hispanic White | 0.85 (0.73, 0.98) | **0.02** | Ref | 0.85 (0.56, 1.29) | 0.44 | 0.66 (0.44, 1.01) | 0.06 | 0.71 (0.46, 1.11) |  |
| Non-Hispanic Black | 1.00 (0.81, 1.24) | 0.97 | Ref | 0.84 (0.45, 1.55) | 0.57 | 1.16 (0.63, 2.13) | 0.62 | 1.06 (0.55, 2.03) | 0.68 |
| Mexican American | 0.70 (0.51, 0.95) | **0.02** | Ref | 0.61 (0.29, 1.25) | 0.17 | 0.49 (0.24, 0.98) | **0.04** | 0.36 (0.13, 1.04) | 0.14 |
| Other | 0.86 (0.66, 1.14) | 0.29 | Ref | 0.84 (0.41, 1.73) | 0.63 | 0.57 (0.25, 1.27) | 0.17 | 0.67 (0.25, 1.76) | 0.32 |
| **Poverty income ratio** |  |  |  |  |  |  |  |  |  |
| ≤ 1.30 | 0.96 (0.79, 1.17) | 0.7 | Ref | 0.86 (0.54, 1.37) | 0.52 | 0.99 (0.60, 1.63) | 0.97 | 0.88 (0.49, 1.61) | **0.04** |
| 1.31 – 3.50 | 0.84 (0.71, 0.98) | **0.03** | Ref | 0.89 (0.52, 1.50) | 0.65 | 0.60 (0.34, 1.08) | 0.09 | 0.67 (0.39, 1.14) | 0.28 |
| > 3.5 | 0.84 (0.66, 1.08) | 0.17 | Ref | 0.65 (0.30, 1.39) | 0.26 | 0.58 (0.29, 1.17) | 0.13 | 0.70 (0.35, 1.41) | 0.59 |
| **Educational attainment** |  |  |  |  |  |  |  |  | 0.09 |
| < High school | 0.83 (0.67, 1.03) | 0.09 | Ref | 0.68 (0.38, 1.24) | 0.21 | 0.80 (0.46, 1.40) | 0.44 | 0.54 (0.30, 0.96) |  |
| ≥ High school | 0.88 (0.77, 1.01) | 0.06 | Ref | 0.85 (0.58, 1.25) | 0.41 | 0.66 (0.45, 0.97) | **0.04** | 0.79 (0.52, 1.21) | 0.13 |
| **BMI** |  |  |  |  |  |  |  |  |  |
| 25 – 30.0 | 0.96 (0.79, 1.16) | 0.66 | Ref | 1.34 (0.68, 2.63) | 0.4 | 0.99 (0.51, 1.93) | 0.99 | 1.23 (0.63, 2.41) | 0.54 |
| < 25 | 0.81 (0.63, 1.04) | 0.1 | Ref | 0.42 (0.22, 0.81) | **0.01** | 0.39 (0.21, 0.75) | **0.005** | 0.56 (0.28, 1.14) | 0.11 |
| ≥ 30.0 | 0.84 (0.73, 0.98) | **0.03** | Ref | 0.89 (0.56, 1.42) | 0.63 | 0.78 (0.48, 1.26) | 0.31 | 0.61 (0.39, 0.96) | **0.03** |
| **Alcohol consumption** |  |  |  |  |  |  |  |  |  |
| Never | 0.88 (0.73, 1.06) | 0.18 | Ref | 1.22 (0.54, 2.73) | 0.63 | 1.22 (0.67, 2.22) | 0.52 | 0.64 (0.30, 1.35) | 0.24 |
| Former | 0.80 (0.63, 1.00) | **0.05** | Ref | 1.06 (0.57, 1.98) | 0.85 | 0.65 (0.34, 1.22) | 0.18 | 0.64 (0.28, 1.47) | 0.29 |
| Mild | 0.95 (0.76, 1.20) | 0.68 | Ref | 1.02 (0.58, 1.80) | 0.95 | 0.97 (0.56, 1.67) | 0.91 | 1.12 (0.60, 2.12) | 0.72 |
| Moderate | 0.84 (0.64, 1.10) | 0.2 | Ref | 0.41 (0.17, 0.99) | **0.05** | 0.57 (0.21, 1.56) | 0.27 | 0.59 (0.20, 1.73) | 0.33 |
| Heavy | 0.85 (0.61, 1.17) | 0.31 | Ref | 0.36 (0.13, 1.02) | 0.06 | 0.26 (0.11, 0.59) | **0.002** | 0.56 (0.24, 1.31) | 0.18 |
| **Physical activity** |  |  |  |  |  |  |  |  |  |
| No | 0.88 (0.75, 1.03) | 0.11 | Ref | 0.93 (0.60, 1.46) | 0.76 | 0.85 (0.55, 1.33) | 0.48 | 0.76 (0.46, 1.24) | 0.26 |
| Yes | 0.84 (0.72, 0.99) | **0.04** | Ref | 0.66 (0.40, 1.10) | 0.11 | 0.50 (0.31, 0.80) | **0.004** | 0.68 (0.44, 1.08) | 0.1 |

Notes: Fully adjusted model, adjusted for age, sex, ethnicity, educational attainment, BMI, poverty income ratio, alcohol consumption and physical activity. Continuous, per doubling concentration of PFNA. CI: confidence interval; OR: odds ratio; Q, quartile; Ref, reference. BMI, body mass index.

Bold values indicate statistical significance (*p* < 0.05).

**Supplemental Table S6.** Subgroup analysis of the association between PFUnDA exposure and odds of RA by the general characteristics of participants in fully adjusted model.

| **Characteristic** | **Continuous** | | **Q1** | **Q2** | | **Q3** | | **Q4** | |
| --- | --- | --- | --- | --- | --- | --- | --- | --- | --- |
|  | **OR (95% CI)** | ***p*** |  | **OR (95% CI)** | ***p*** | **OR (95% CI)** | ***p*** | **OR (95% CI)** | ***p*** |
| **Age** |  |  |  |  |  |  |  |  |  |
| 20 – 39 | 0.92 (0.71, 1.19) | 0.5 | Ref | 1.17 (0.47, 2.90) | 0.73 | 1.85 (0.84, 4.09) | 0.13 | 0.09 (0.01, 0.75) | **0.03** |
| 40 – 59 | 0.85 (0.70, 1.02) | 0.08 | Ref | 0.76 (0.48, 1.20) | 0.23 | 0.70 (0.43, 1.16) | 0.16 | 0.70 (0.39, 1.26) | 0.23 |
| ≥ 60 | 0.97 (0.84, 1.12) | 0.69 | Ref | 0.91 (0.56, 1.48) | 0.71 | 0.80 (0.54, 1.20) | 0.28 | 0.81 (0.51, 1.29) | 0.36 |
| **Sex** |  |  |  |  |  |  |  |  |  |
| Male | 1.05 (0.91, 1.22) | 0.51 | Ref | 0.86 (0.48, 1.52) | 0.59 | 0.98 (0.59, 1.61) | 0.93 | 1.02 (0.59, 1.77) | 0.95 |
| Female | 0.81 (0.71, 0.93) | **0.004** | Ref | 0.86 (0.60, 1.25) | 0.43 | 0.73 (0.50, 1.07) | 0.1 | 0.56 (0.36, 0.86) | **0.01** |
| **Ethnicity** |  |  |  |  |  |  |  |  |  |
| Non-Hispanic White | 0.88 (0.75, 1.02) | 0.09 | Ref | 0.87 (0.58, 1.29) | 0.49 | 0.69 (0.46, 1.03) | 0.07 | 0.69 (0.43, 1.10) | 0.12 |
| Non-Hispanic Black | 1.01 (0.88, 1.16) | 0.9 | Ref | 0.87 (0.46, 1.65) | 0.67 | 1.05 (0.61, 1.79) | 0.86 | 1.06 (0.61, 1.84) | 0.83 |
| Mexican American | 0.95 (0.64, 1.41) | 0.78 | Ref | 0.84 (0.35, 1.99) | 0.69 | 1.42 (0.60, 3.37) | 0.42 | 0.21 (0.04, 1.01) | **0.05** |
| Other | 0.88 (0.69, 1.11) | 0.28 | Ref | 0.84 (0.35, 2.03) | 0.7 | 1.53 (0.64, 3.66) | 0.34 | 0.54 (0.23, 1.31) | 0.17 |
| **Poverty income ratio** |  |  |  |  |  |  |  |  |  |
| ≤ 1.30 | 0.92 (0.77, 1.08) | 0.3 | Ref | 1.00 (0.58, 1.74) | 0.99 | 0.99 (0.57, 1.70) | 0.96 | 0.71 (0.40, 1.28) | 0.25 |
| 1.31 – 3.50 | 0.88 (0.74, 1.04) | 0.13 | Ref | 0.97 (0.56, 1.69) | 0.92 | 1.03 (0.64, 1.66) | 0.9 | 0.52 (0.29, 0.95) | **0.03** |
| > 3.5 | 0.96 (0.76, 1.22) | 0.73 | Ref | 0.64 (0.33, 1.25) | 0.19 | 0.47 (0.25, 0.88) | **0.02** | 0.90 (0.47, 1.71) | 0.74 |
| **Educational attainment** |  |  |  |  |  |  |  |  |  |
| < High school | 0.90 (0.75, 1.07) | 0.23 | Ref | 0.84 (0.50, 1.42) | 0.51 | 0.80 (0.47, 1.38) | 0.42 | 0.68 (0.39, 1.20) | 0.19 |
| ≥ High school | 0.92 (0.81, 1.04) | 0.17 | Ref | 0.86 (0.58, 1.28) | 0.47 | 0.81 (0.55, 1.20) | 0.29 | 0.75 (0.50, 1.11) | 0.15 |
| **BMI** |  |  |  |  |  |  |  |  |  |
| 25 – 30.0 | 0.87 (0.70, 1.10) | 0.25 | Ref | 0.71 (0.42, 1.21) | 0.2 | 0.58 (0.32, 1.05) | 0.07 | 0.73 (0.36, 1.48) | 0.37 |
| < 25 | 0.90 (0.73, 1.11) | 0.34 | Ref | 1.16 (0.53, 2.55) | 0.71 | 1.19 (0.54, 2.63) | 0.66 | 0.60 (0.28, 1.30) | 0.19 |
| ≥ 30.0 | 0.94 (0.81, 1.09) | 0.41 | Ref | 0.84 (0.53, 1.34) | 0.46 | 0.82 (0.54, 1.25) | 0.35 | 0.83 (0.49, 1.42) | 0.5 |
| **Alcohol consumption** |  |  |  |  |  |  |  |  |  |
| Never | 0.79 (0.62, 1.00) | **0.05** | Ref | 0.90 (0.45, 1.80) | 0.77 | 0.64 (0.33, 1.23) | 0.18 | 0.60 (0.29, 1.24) | 0.17 |
| Former | 0.87 (0.70, 1.09) | 0.24 | Ref | 0.73 (0.39, 1.36) | 0.32 | 0.78 (0.40, 1.50) | 0.45 | 0.50 (0.25, 0.98) | **0.04** |
| Mild | 0.97 (0.82, 1.15) | 0.75 | Ref | 0.95 (0.49, 1.83) | 0.88 | 0.84 (0.50, 1.44) | 0.53 | 1.01 (0.56, 1.83) | 0.97 |
| Moderate | 1.01 (0.74, 1.37) | 0.96 | Ref | 1.52 (0.42, 5.43) | 0.52 | 1.69 (0.49, 5.91) | 0.41 | 1.05 (0.29, 3.87) | 0.94 |
| Heavy | 0.89 (0.61, 1.29) | 0.53 | Ref | 0.67 (0.28, 1.61) | 0.37 | 0.61 (0.23, 1.59) | 0.31 | 0.57 (0.22, 1.45) | 0.24 |
| **Physical activity** |  |  |  |  |  |  |  |  |  |
| No | 0.92 (0.80, 1.05) | 0.2 | Ref | 1.06 (0.73, 1.53) | 0.76 | 0.75 (0.51, 1.10) | 0.14 | 0.78 (0.49, 1.22) | 0.27 |
| Yes | 0.90 (0.77, 1.05) | 0.19 | Ref | 0.59 (0.32, 1.06) | 0.07 | 0.85 (0.50, 1.44) | 0.54 | 0.64 (0.36, 1.13) | 0.13 |

Notes: Fully adjusted model, adjusted for age, sex, ethnicity, educational attainment, BMI, poverty income ratio, alcohol consumption and physical activity. Continuous, per doubling concentration of PFUnDA. CI: confidence interval; OR: odds ratio; Q, quartile; Ref, reference. BMI, body mass index.

Bold values indicate statistical significance (*p* < 0.05).

**Supplemental Table S7.** Subgroup analysis of the association between Me-PFOSA-AcOH exposure and odds of RA by the general characteristics of participants in fully adjusted model.

| **Characteristic** | **Continuous** | | **Q1** | **Q2** | | **Q3** | | **Q4** | |
| --- | --- | --- | --- | --- | --- | --- | --- | --- | --- |
|  | **OR (95% CI)** | ***p*** |  | **OR (95% CI)** | ***p*** | **OR (95% CI)** | ***p*** | **OR (95% CI)** | ***p*** |
| **Age** |  |  |  |  |  |  |  |  |  |
| 20 – 39 | 1.26 (0.98, 1.62) | 0.07 | Ref | 2.67 (0.88, 8.13) | 0.08 | 1.43 (0.41, 4.94) | 0.57 | 2.63 (0.87, 7.92) | 0.09 |
| 40 – 59 | 1.08 (0.94, 1.24) | 0.3 | Ref | 1.19 (0.73, 1.93) | 0.47 | 1.72 (1.04, 2.86) | **0.04** | 1.17 (0.65, 2.11) | 0.6 |
| ≥ 60 | 1.02 (0.91, 1.15) | 0.69 | Ref | 0.97 (0.59, 1.62) | 0.92 | 1.03 (0.65, 1.63) | 0.9 | 1.07 (0.66, 1.75) | 0.78 |
| **Sex** |  |  |  |  |  |  |  |  |  |
| Male | 1.08 (0.96, 1.22) | 0.21 | Ref | 1.34 (0.68, 2.61) | 0.4 | 1.84 (1.00, 3.38) | **0.05** | 1.27 (0.66, 2.44) | 0.48 |
| Female | 1.03 (0.93, 1.15) | 0.54 | Ref | 1.19 (0.83, 1.69) | 0.34 | 1.12 (0.74, 1.71) | 0.59 | 1.19 (0.79, 1.78) | 0.41 |
| **Ethnicity** |  |  |  |  |  |  |  |  |  |
| Non-Hispanic White | 1.06 (0.96, 1.17) | 0.28 | Ref | 1.30 (0.80, 2.10) | 0.29 | 1.43 (0.88, 2.31) | 0.14 | 1.24 (0.76, 2.04) | 0.39 |
| Non-Hispanic Black | 1.01 (0.87, 1.18) | 0.88 | Ref | 1.04 (0.67, 1.62) | 0.86 | 1.13 (0.65, 1.95) | 0.67 | 1.09 (0.60, 1.97) | 0.79 |
| Mexican American | 1.04 (0.83, 1.30) | 0.73 | Ref | 1.03 (0.50, 2.10) | 0.94 | 1.73 (0.78, 3.84) | 0.18 | 0.90 (0.36, 2.26) | 0.83 |
| Other | 1.14 (0.90, 1.43) | 0.28 | Ref | 1.63 (0.79, 3.37) | 0.18 | 1.15 (0.43, 3.13) | 0.78 | 1.43 (0.64, 3.18) | 0.38 |
| **Poverty income ratio** |  |  |  |  |  |  |  |  |  |
| ≤ 1.30 | 1.09 (0.98, 1.22) | 0.12 | Ref | 1.43 (0.94, 2.17) | 0.1 | 1.83 (1.10, 3.05) | **0.02** | 1.39 (0.85, 2.29) | 0.19 |
| 1.31 – 3.50 | 1.11 (0.98, 1.25) | 0.1 | Ref | 1.64 (0.92, 2.93) | 0.09 | 1.41 (0.78, 2.54) | 0.25 | 1.63 (0.91, 2.92) | 0.1 |
| > 3.5 | 0.97 (0.82, 1.14) | 0.69 | Ref | 0.83 (0.46, 1.50) | 0.52 | 1.10 (0.57, 2.13) | 0.78 | 0.79 (0.39, 1.62) | 0.52 |
| **Educational attainment** |  |  |  |  |  |  |  |  |  |
| < High school | 0.98 (0.87, 1.11) | 0.79 | Ref | 1.06 (0.64, 1.78) | 0.81 | 1.05 (0.63, 1.74) | 0.85 | 0.77 (0.43, 1.38) | 0.38 |
| ≥ High school | 1.07 (0.98, 1.18) | 0.13 | Ref | 1.28 (0.87, 1.88) | 0.21 | 1.46 (0.96, 2.23) | 0.08 | 1.36 (0.90, 2.05) | 0.15 |
| **BMI** |  |  |  |  |  |  |  |  |  |
| 25 – 30.0 | 1.08 (0.94, 1.24) | 0.29 | Ref | 1.23 (0.66, 2.27) | 0.51 | 1.19 (0.59, 2.40) | 0.63 | 1.59 (0.82, 3.06) | 0.17 |
| < 25 | 1.09 (0.92, 1.28) | 0.32 | Ref | 1.61 (0.67, 3.88) | 0.28 | 2.12 (0.84, 5.31) | 0.11 | 1.67 (0.69, 4.06) | 0.25 |
| ≥ 30.0 | 1.00 (0.90, 1.12) | 0.97 | Ref | 1.14 (0.72, 1.82) | 0.57 | 1.29 (0.81, 2.04) | 0.28 | 0.80 (0.51, 1.26) | 0.34 |
| **Alcohol consumption** |  |  |  |  |  |  |  |  |  |
| Never | 1.08 (0.93, 1.26) | 0.3 | Ref | 0.96 (0.45, 2.06) | 0.91 | 0.86 (0.38, 1.98) | 0.72 | 1.18 (0.61, 2.26) | 0.62 |
| Former | 0.98 (0.83, 1.16) | 0.82 | Ref | 1.47 (0.68, 3.17) | 0.33 | 1.15 (0.54, 2.45) | 0.72 | 1.02 (0.48, 2.17) | 0.96 |
| Mild | 1.07 (0.95, 1.22) | 0.27 | Ref | 1.59 (0.87, 2.94) | 0.13 | 1.91 (0.99, 3.70) | **0.05** | 1.30 (0.68, 2.48) | 0.42 |
| Moderate | 1.19 (0.98, 1.45) | 0.09 | Ref | 0.91 (0.34, 2.40) | 0.84 | 1.54 (0.61, 3.84) | 0.36 | 2.11 (0.89, 5.00) | 0.09 |
| Heavy | 1.05 (0.81, 1.36) | 0.71 | Ref | 0.84 (0.32, 2.22) | 0.72 | 1.36 (0.52, 3.51) | 0.53 | 1.08 (0.41, 2.89) | 0.87 |
| **Physical activity** |  |  |  |  |  |  |  |  |  |
| No | 1.05 (0.95, 1.16) | 0.32 | Ref | 1.31 (0.88, 1.95) | 0.18 | 1.14 (0.72, 1.82) | 0.57 | 1.28 (0.84, 1.96) | 0.25 |
| Yes | 1.05 (0.94, 1.18) | 0.4 | Ref | 1.09 (0.63, 1.91) | 0.75 | 1.68 (1.03, 2.74) | **0.04** | 1.07 (0.61, 1.89) | 0.82 |

Notes: Fully adjusted model, adjusted for age, sex, ethnicity, educational attainment, BMI, poverty income ratio, alcohol consumption and physical activity. Continuous, per doubling concentration of Me-PFOSA-AcOH. CI: confidence interval; OR: odds ratio; Q, quartile; Ref, reference. BMI, body mass index.

Bold values indicate statistical significance (*p* < 0.05).

**Supplemental Table S8.** Sensitivity analysis of the association between PFAS exposure and odds of RA through additionally adjusting the NHANES calendar cycle.

| **PFAS** | **Continuous** | | **Q1** | **Q2** | | **Q3** | | **Q4** | |
| --- | --- | --- | --- | --- | --- | --- | --- | --- | --- |
| **(ng/mL)** | **OR (95% CI)** | ***p*** |  | **OR (95% CI)** | ***p*** | **OR (95% CI)** | ***p*** | **OR (95% CI)** | ***p*** |
| **PFOA** | 0.85 (0.76, 0.95) | **0.004** | Ref | 0.65 (0.44, 0.94) | **0.02** | 0.79 (0.56, 1.12) | 0.18 | 0.52 (0.37, 0.75) | **<0.001** |
| **PFOS** | 0.94 (0.85, 1.03) | 0.18 | Ref | 0.81 (0.57, 1.15) | 0.23 | 0.86 (0.59, 1.24) | 0.41 | 0.70 (0.46, 1.08) | 0.11 |
| **PFHxS** | 0.90 (0.82, 0.99) | **0.03** | Ref | 0.77 (0.57, 1.05) | 0.1 | 0.69 (0.48, 0.99) | **0.04** | 0.68 (0.48, 0.98) | **0.04** |
| **PFDA** | 0.88 (0.79, 0.99) | **0.03** | Ref | 0.88 (0.62, 1.25) | 0.47 | 0.80 (0.59, 1.09) | 0.16 | 0.70 (0.50, 0.97) | **0.03** |
| **PFNA** | 0.86 (0.76, 0.97) | **0.02** | Ref | 0.80 (0.57, 1.13) | 0.2 | 0.69 (0.50, 0.93) | **0.02** | 0.72 (0.49, 1.04) | 0.08 |
| **PFUnDA** | 0.88 (0.80, 0.97) | **0.01** | Ref | 0.81 (0.56, 1.16) | 0.25 | 0.60 (0.43, 0.83) | **0.002** | 0.68 (0.48, 0.94) | **0.02** |
| **Me-PFOSA-AcOH** | 1.06 (0.97, 1.15) | 0.2 | Ref | 1.30 (0.93, 1.80) | 0.12 | 1.37 (0.91, 2.06) | 0.13 | 1.29 (0.90, 1.85) | 0.17 |

Notes: adjusted for age, sex, ethnicity, educational attainment, BMI, poverty income ratio, alcohol consumption, physical activity and NHANES calendar cycle. Continuous, per doubling concentration of PFAS; CI: confidence interval; OR: odds ratio; Q, quartile; Ref, reference; Bold values indicate statistical significance (*p* < 0.05).

**Supplemental Table S9.** The multi-cycle sensitivity analysis of the association between PFAS exposure and odds of RA.

| **NHANES cycle** | **PFOA** | | **PFOS** | | **PFHxS** | | **PFDA** | | **PFNA** | | **PFUnDA** | | **Me-PFOSA-AcOH** | |
| --- | --- | --- | --- | --- | --- | --- | --- | --- | --- | --- | --- | --- | --- | --- |
|  | **OR (95% CI)** | ***p*** | **OR (95% CI)** | ***p*** | **OR (95% CI)** | ***p*** | **OR (95% CI)** | ***p*** | **OR (95% CI)** | ***p*** | **OR (95% CI)** | ***p*** | **OR (95% CI)** | ***p*** |
| 2003-2004 | 1.06 (0.85, 1.33) | 0.57 | 1.12 (0.89, 1.41) | 0.33 | 1.07 (0.89, 1.28) | 0.46 | 1.11 (0.80, 1.53) | 0.52 | 1.19 (0.88, 1.61) | 0.25 | 0.95 (0.73, 1.24) | 0.7 | 1.08 (0.66, 1.75) | 0.75 |
| 2005-2006 | 0.97 (0.76, 1.23) | 0.76 | 0.97 (0.75, 1.25) | 0.78 | 1.03 (0.85, 1.24) | 0.78 | 0.90 (0.62, 1.32) | 0.58 | 0.90 (0.66, 1.23) | 0.5 | 0.88 (0.61, 1.28) | 0.48 | 1.35 (1.20, 1.50) | **<0.0001** |
| 2007-2008 | 0.63 (0.42, 0.94) | 0.25 | 0.76 (0.63, 0.93) | 0.21 | 0.87 (0.71, 1.08) | 0.4 | 0.77 (0.54, 1.09) | 0.36 | 0.65 (0.46, 0.90) | 0.22 | 0.82 (0.55, 1.23) | 0.49 | 0.78 (0.59, 1.03) | 0.31 |
| 2009-2010 | 0.95 (0.71, 1.27) | 0.77 | 1.04 (0.83, 1.31) | 0.77 | 0.84 (0.65, 1.09) | 0.39 | 0.83 (0.65, 1.05) | 0.34 | 0.92 (0.68, 1.24) | 0.67 | 0.85 (0.70, 1.04) | 0.33 | 1.05 (0.86, 1.29) | 0.7 |
| 2011-2012 | 0.90 (0.48, 1.68) | 0.53 | 1.06 (0.52, 2.17) | 0.74 | 0.88 (0.51, 1.52) | 0.42 | 0.87 (0.44, 1.74) | 0.49 | 1.00 (0.40, 2.48) | 0.83 | 1.18 (0.67, 2.11) | 0.33 | 1.32 (0.86, 2.02) | 0.11 |
| 2013-2014 | 0.84 (0.60, 1.17) | 0.28 | 0.91 (0.73, 1.13) | 0.37 | 0.90 (0.60, 1.34) | 0.57 | 0.91 (0.74, 1.12) | 0.35 | 0.69 (0.57, 0.83) | **<0.001** | 0.77 (0.52, 1.14) | 0.17 | 1.02 (0.73, 1.42) | 0.9 |
| 2015-2016 | 0.66 (0.51, 0.85) | **0.004** | 0.75 (0.60, 0.95) | **0.02** | 0.75 (0.58, 0.98) | **0.03** | 0.81 (0.59, 1.11) | 0.17 | 0.69 (0.48, 0.98) | **0.04** | 0.83 (0.63, 1.09) | 0.17 | 0.87 (0.71, 1.07) | 0.18 |
| 2017-2018 | 0.77 (0.47, 1.28) | 0.47 | 1.18 (0.67, 2.05) | 0.64 | 0.86 (0.60, 1.22) | 0.52 | 1.01 (0.60, 1.69) | 0.98 | 0.87 (0.60, 1.28) | 0.59 | 0.85 (0.54, 1.34) | 0.59 | 0.94 (0.69, 1.29) | 0.76 |
| Combined | 0.87(0.78,0.97) | - | 0.91 (0.83, 1.00) | - | 0.93(0.85, 1.02) | - | 0.89 (0.79, 0.99) | - | 0.81 (0.72, 0.90) | - | 0.87 (0.78, 0.97) | - | 1.04 (0.90, 1.20) | - |

Notes: All individual NHANES cycle study was weighted by their own 2 years subsample weight; OR: odds ratio per doubling concentration of PFAS; CI: confidence interval; PFOA: perfluorooctanoic acid; PFOS: perfluorooctane sulfonate; PFHxS: perfluorohexane sulfonic acid; PFDA: perfluorodecanoic acid; PFNA: perfluorononanoic acid; PFUnDA: perfluoroundecanoic acid; Me-PFOSA-AcOH: methyl perfluorooctane sulfonamidoacetic acid.

Bold values indicate statistical significance (*p* < 0.05).

**Supplemental Fig. S1.** Directed Acyclic Graph for covariate selection.


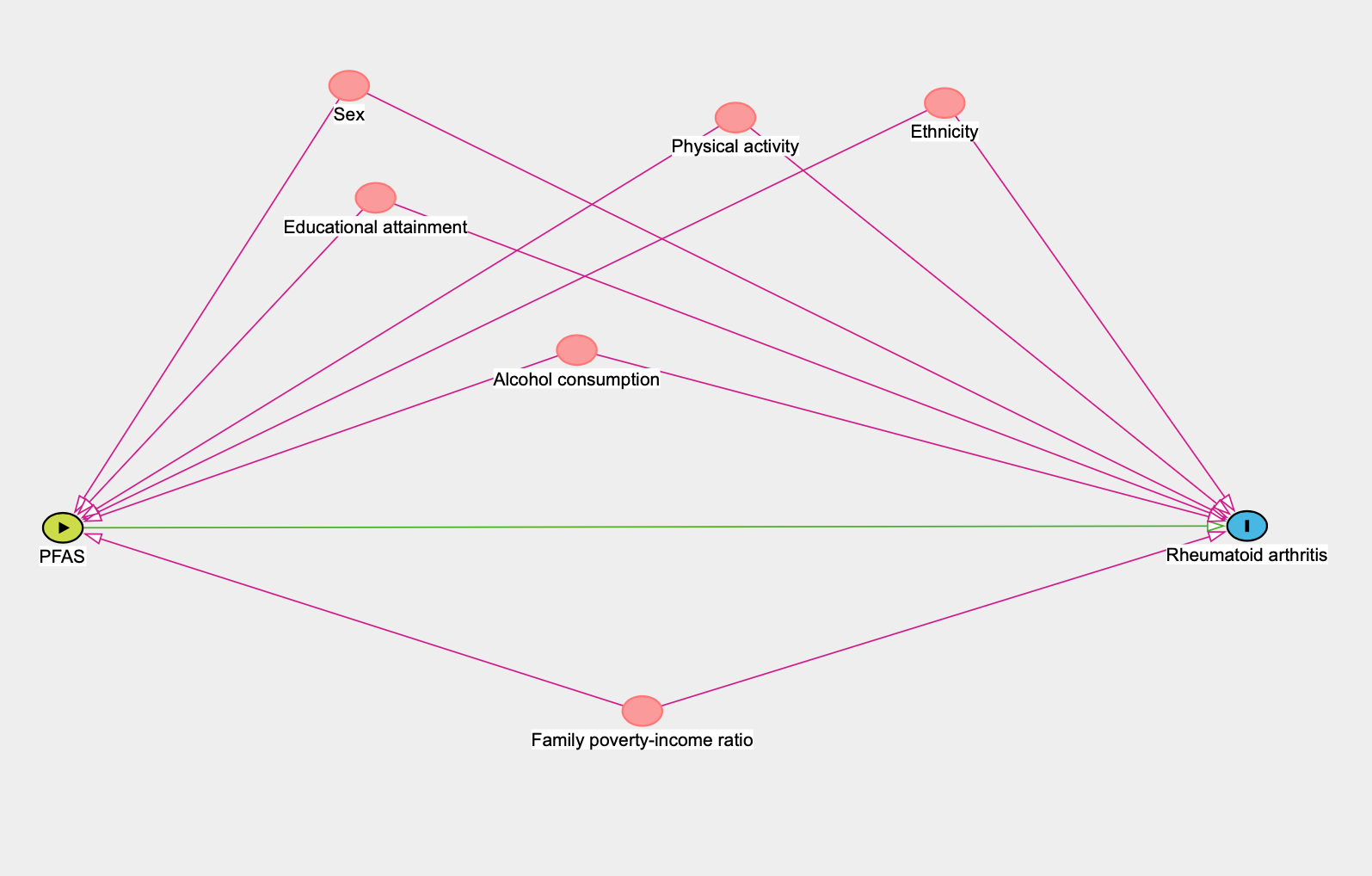


**Supplemental Fig. S2.** Univariate exposure-response functions for RA risk from the BKMR model. Associations between each PFAS and RA risk (with corresponding 95% credible intervals) are shown when setting all other PFASs at their median. Models adjusted for age, sex, ethnicity, educational attainment, body mass index, poverty income ratio, alcohol consumption and physical activity.


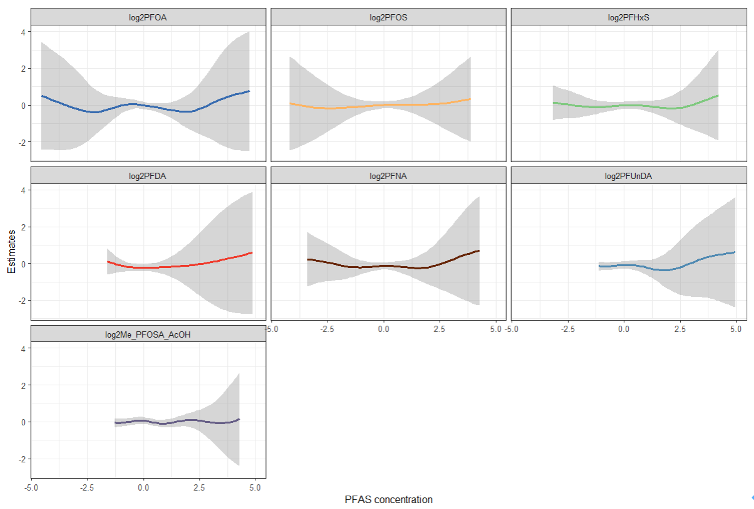


**Supplemental Fig. S3.** The joint effect of the PFAS mixtures on RA risk estimated from the BKMR model. Estimates and 95% credible intervals are shown in the figure when all exposures at particular percentiles were compared to all the congeners at their 50th percentile. Models adjusted for age, sex, ethnicity, educational attainment, body mass index, poverty income ratio, alcohol consumption and physical activity.


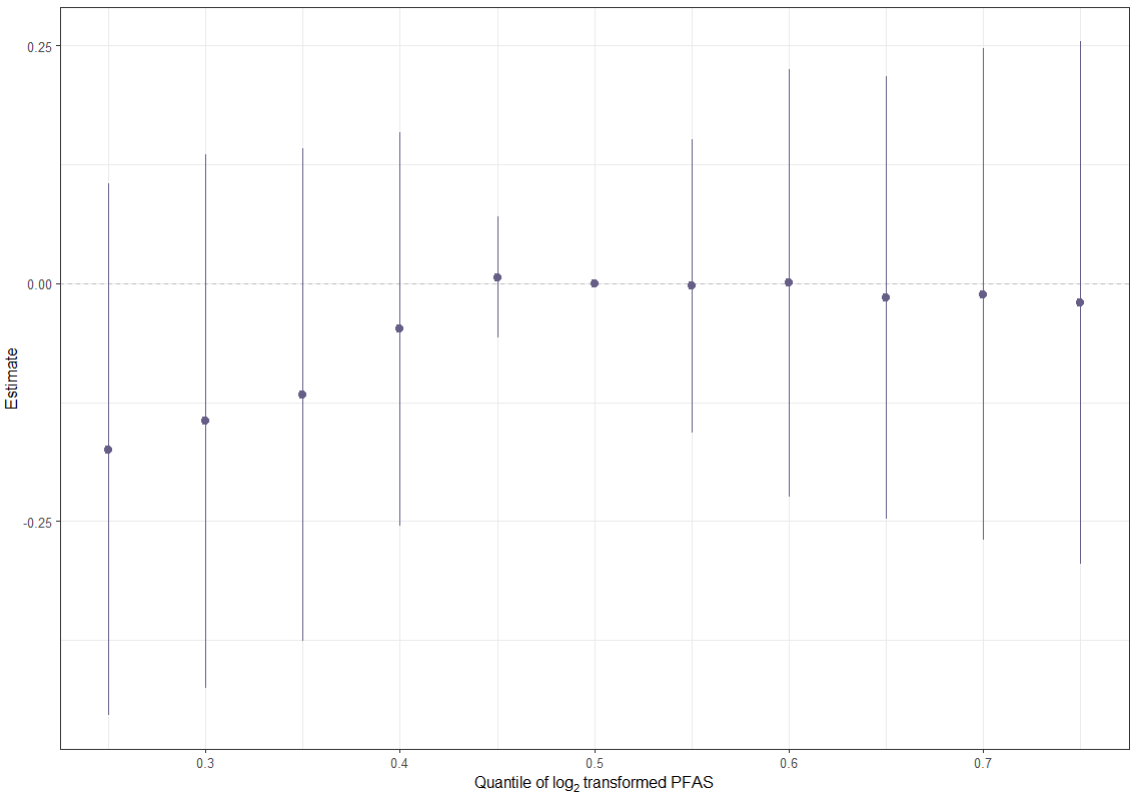


**Supplemental Fig. S4.** Associations of each individual PFAS with RA status in BKMR model. This plot describes the estimated RA status associated with a change in each individual PFAS from its 25th to 75th percentile, when all the other PFAS are fixed at either the 25th (red line), 50th (green line), or 75th percentile (blue line). Dots indicate the estimate, and horizontal lines indicate the 95% credible intervals. All models were adjusted for age, sex, ethnicity, educational attainment, body mass index, poverty income ratio, alcohol consumption and physical activity.


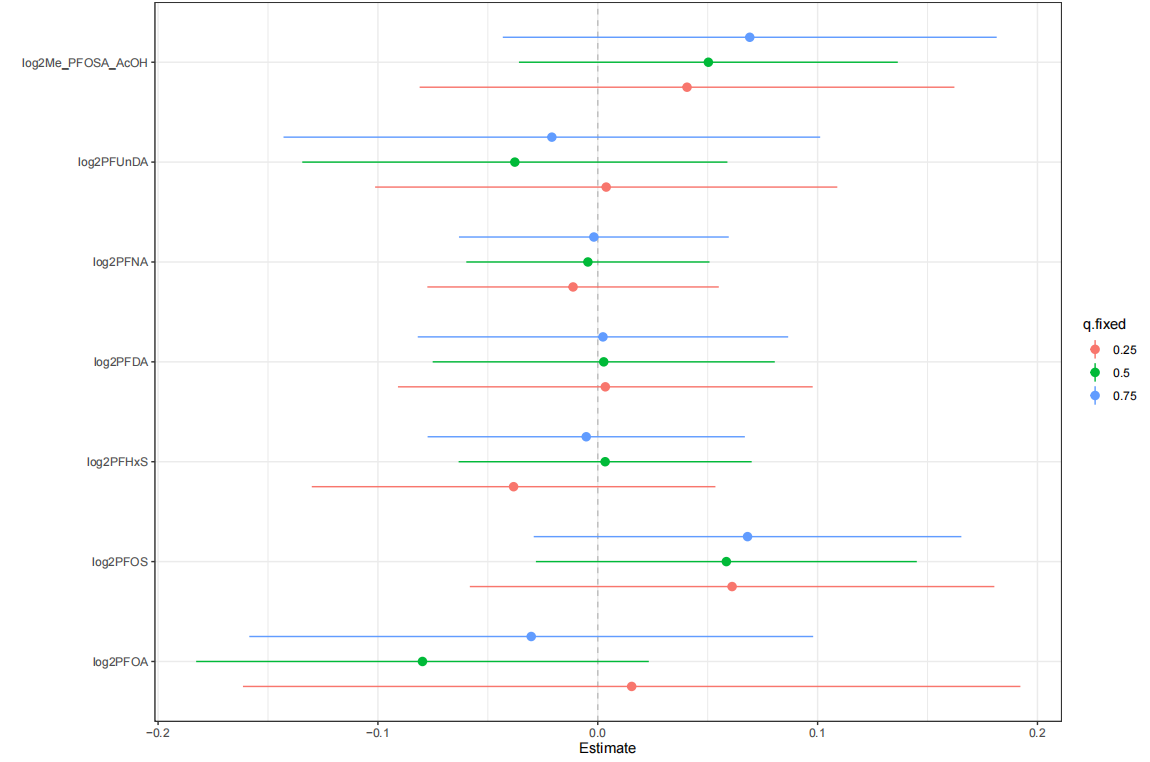


**Supplemental Fig. S5.** Univariate exposure-response functions for RA risk from the BKMR model stratified by sex. Associations between each PFAS and RA risk (with corresponding 95% credible intervals) are shown when setting all other PFASs at their median. Models adjusted for age, sex, ethnicity, educational attainment, body mass index, poverty income ratio, alcohol consumption and physical activity.


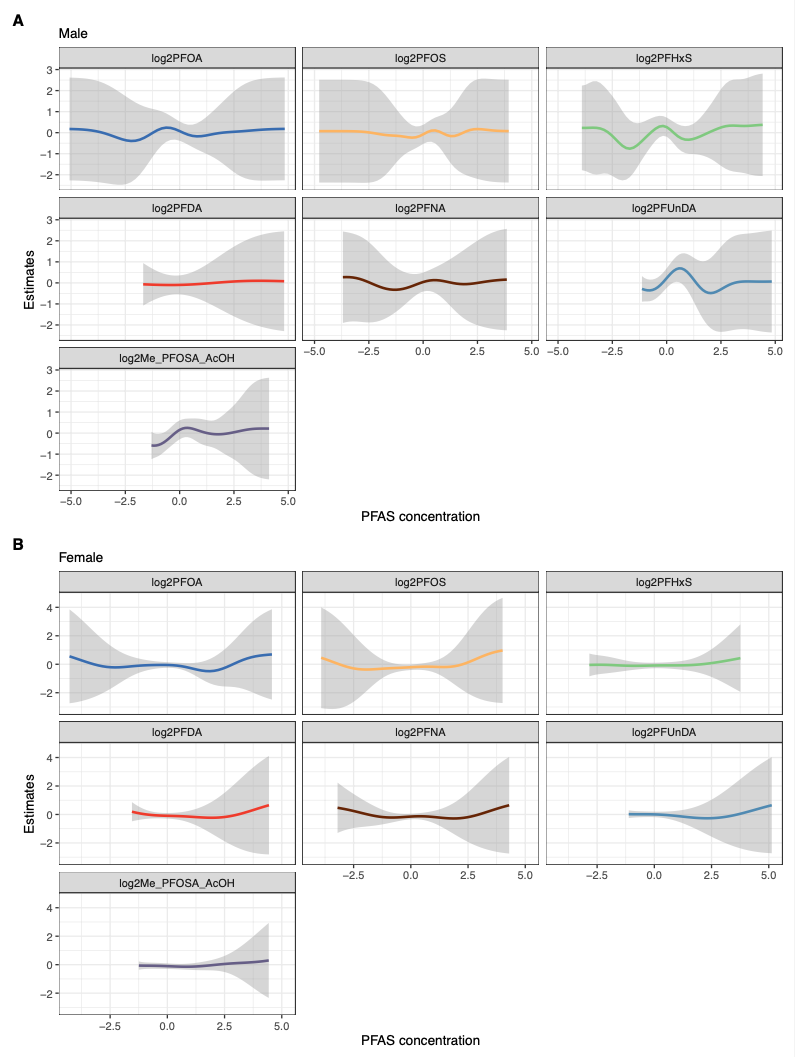


**Supplemental Fig. S6.** The joint effect of the PFAS mixtures on RA risk estimated from the BKMR model stratified by sex. Estimates and 95% credible intervals are shown in the figure when all exposures at particular percentiles were compared to all the congeners at their 50th percentile. Models adjusted for age, sex, ethnicity, educational attainment, body mass index, poverty income ratio, alcohol consumption and physical activity.

**Supplemental Fig. S7.** Association between per- and polyfluoroalkyl substances (PFAS) composite levels and rheumatoid arthritis (RA) status based on weighted quantile sum (WQS) regression analysis. Two separate WQS indices were generated, one modeled in the positive direction and one modeled in the inverse direction with respect to RA status.


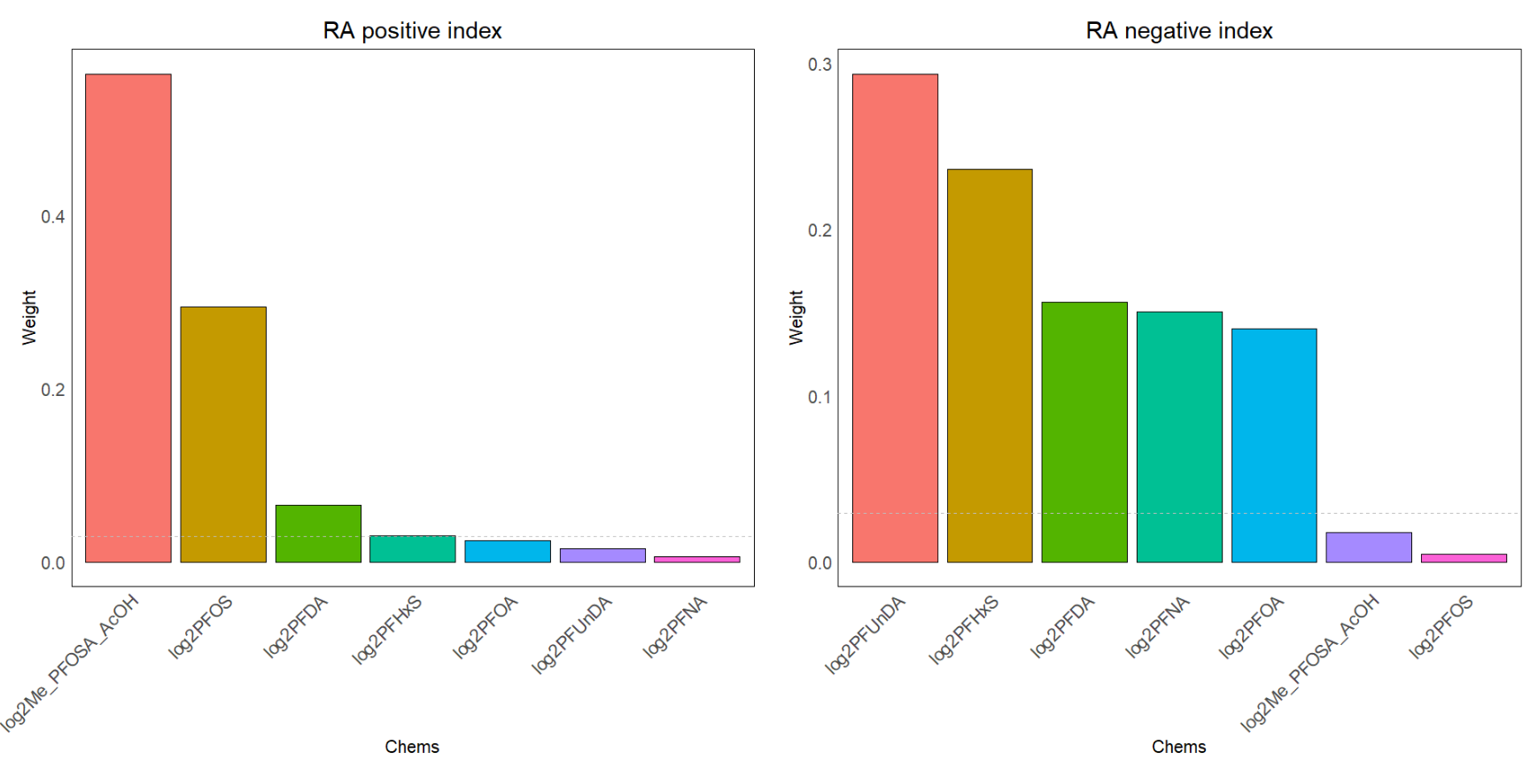


**Supplemental Fig. S8.** Mean adjusted ORs (A) and sex-specific relative weights (B) from a WQS logistic regression model with 100 repeated holdouts between PFAS mixture analysis and odds of RA. The model was adjusted for age, sex, ethnicity, educational attainment, BMI, poverty income ratio, alcohol consumption and physical activity. (A) Illustrates the distribution of the adjusted ORs across the 100 repeated holdouts where each dot represents the estimate from each holdout. (B) Illustrates the mean estimated relative weight for each chemical of the PFAS mixture across the 100 repeated holdouts. The relative weight is the percentage of weight attributable to each chemical in the PFAS mixture within the total weight of each strata (male and female). The dashed line represents the threshold (14.29%) for chemicals of concern. Chemicals with relative weights above this threshold in at least 50% of the repeated holdouts were considered chemicals of concern. Notes: All chemicals were log_2_ transformed to reduce skewness in the distribution of the concentrations.
